# Microscopic mechanism of biphasic interface relaxation in lithium iron phosphate after delithiation

**DOI:** 10.1038/s41467-018-05241-1

**Published:** 2018-07-20

**Authors:** Shunsuke Kobayashi, Akihide Kuwabara, Craig A. J. Fisher, Yoshio Ukyo, Yuichi Ikuhara

**Affiliations:** 1 Nanostructures Research Laboratory, Japan Fine Ceramics Center, Atsuta, Nagoya, 456-8587 Japan; 2 Office of Society-Academia Collaboration for Innovation, Kyoto University, Uji, Kyoto, 611-0011 Japan; 3 Institute of Engineering Innovation, The University of Tokyo, Bunkyo, Tokyo, 113-8656 Japan

## Abstract

Charge/discharge of lithium-ion battery cathode material LiFePO_4_ is mediated by the structure and properties of the interface between delithiated and lithiated phases. Direct observations of the interface in a partially delithiated single crystal as a function of time using scanning transmission electron microscopy and electron energy-loss spectroscopy help clarify these complex phenomena. At the nano-scale, the interface comprises a thin multiphase layer whose composition varies monotonically between those of the two end-member phases. After partial delithiation, the interface does not remain static, but changes gradually in terms of orientation, morphology and position, as Li ions from the crystal bulk diffuse back into the delithiated regions. First-principles calculations of a monoclinic crystal of composition Li_2/3_FePO_4_ suggest that the interface exhibits higher electronic conductivity than either of the end-member phases. These observations highlight the importance of the interface in enabling LiFePO_4_ particles to retain structural integrity during high-rate charging and discharging.

## Abstract (web summary)

Improving the performance of Li-ion batteries relies on understanding charging/discharging mechanisms. Here the authors visualize the interfacial structure and composition of a partially delithiated lithium iron phosphate single crystal as a function of time, revealing a mechanism of relaxation.

## Introduction

Improving the charge performance of Li-ion batteries for a wide spectrum of applications from mobile devices to electric vehicles is a major goal of contemporary materials research. Charge performance is typically limited by the rate of charge/discharge that can be sustained by the positive electrode without serious degradation over many battery cycles, so the development of structurally and chemically stable positive electrode materials is crucial for achieving this goal. Olivine-structured lithium iron phosphate, LiFePO_4_, first reported in 1997 by Goodenough and coworkers^1^, is a positive electrode material with good stability and cyclability that continues to be intensively studied^2–5^. Nano-sizing^6^ and carbon-coating^7,8^ are well-established techniques for overcoming the poor intrinsic electronic and ionic conductivities of LiFePO_4_^9^, but to improve its performance further requires better understanding of lithium (de)intercalation and related mechanisms at the atomic level; because these processes normally occur via a two-phase reaction in olivine-structured LiFePO_4_, this necessarily includes examining the interface between the lithiated (Li-rich; Li_1-*α*_FePO_4_, *α* < 0.2) and delithiated (Li-poor; Li_*β*_FePO_4_, *β* < 0.2) phases within particles as charging/discharging proceeds.

Recent studies have confirmed that a metastable phase with intermediate Li content forms between Li-rich and Li-poor phases when the material is charged under far-from-equilibrium conditions^10,11^ or at high-charging rates^12,13^. Both end-member phases have olivine-type crystal structures belonging to orthorhombic space group *Pnma*, although there is considerable lattice mismatch between them because of the large volume contraction when Li is removed from the crystal^14^. Formation of an intermediate phase is thought to reduce the excessive lattice strain that would otherwise exist if the end-member phases met at an atomically abrupt interface. This intermediate phase has been investigated recently using theoretical^15–17^ and experimental^11–13,18–20^ approaches, but many aspects remain unknown.

Two powerful methods for investigating the local interface structure and morphologies of complex crystalline systems are scanning transmission electron microscopy (STEM)^21–27^ and electron energy-loss spectroscopy (EELS)^28–30^. In this study, we have used these techniques to examine LiFePO_4_ in different states of delithiation at the atomic and nano-scales to gain a better understanding of the structure, chemistry and dynamics of biphasic boundaries. Because the (010) surface of LiFePO_4_ is perpendicular to the main Li-ion migration pathway in the olivine structure, it is considered to be the most pertinent in terms of electrochemical behaviour^31,32^, and this is the surface that we focus on here. Previous reports showed that microcracks form parallel to the (100) plane in LiFePO_4_ particles during delithiation^33^, so observation down the [001] zone axis (parallel to any cracks and perpendicular to the direction of Li migration) provides an unobstructed view of the biphase boundary formed by delithiation from (010) surfaces.

In this study, a pristine (001) surface is prepared by cleaving a large (millimetre-size) single crystal. STEM observations confirmed it to be atomically flat over a wide area^34^, as shown in Supplementary Note 1 and  Supplementary Fig. 1, making it an excellent model for examining fundamental structural and chemical features of LiFePO_4_ both before and after delithiation. Detailed STEM and EELS observations of the interface between Li_1–*α*_FePO_4_ and Li_*β*_FePO_4_ phases at different times after chemical delithiation of this surface reveal that LiFePO_4_ comprises a thin multiphase layer that does not remain static but relaxes gradually outwards to the crystal surface as Li ions diffuse from the crystal bulk back into the delithiated regions. First-principles calculations of a monoclinic crystal of composition Li_2/3_FePO_4_ suggest that the intermediate phases comprising the interface exhibit higher electronic conductivity than the end-member phases. The findings not only offer fresh insights into the role of the interface layer but also have implications for the development of olivine-type cathode materials and other topotactic materials.

## Results

### Morphology of the boundary between LiFePO_4_ and FePO_4_

**Fig. 1:**
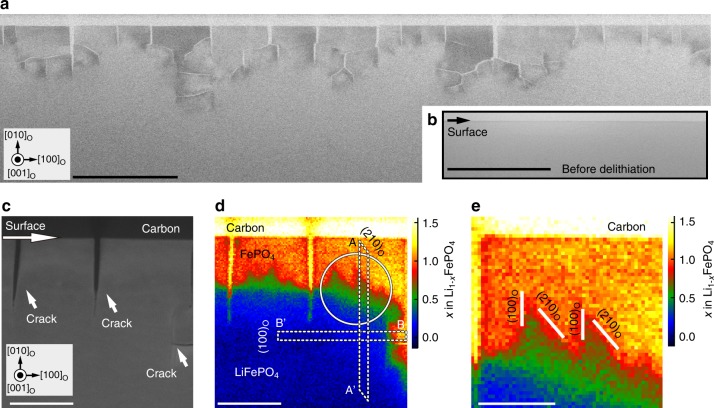
Boundary region between LiFePO_4_ and FePO_4_ after chemical delithiation. **a**, **b** Low-magnification BF STEM images of a (010) surface (**a**) after and (**b**) before delithiation. Subscript “o” refers to the orthorhombic structure. Scale bar, 500 nm. **c** ADF STEM image of a section of the surface in **a**. Scale bar, 100 nm. **d** Li concentration map from the same region as **c**. Scale bar, 100 nm. **e** Magnified Li concentration map of circled region in **d**. Scale bar, 50 nm

Figure 1a, b shows low-magnification bright-field (BF) STEM images of the (010) surface after and before chemical delithiation, respectively. After delithiation, several microcracks formed parallel to the (100) plane in response to the strain induced by large differences in molar volume between delithiated and lithiated phases^25,35^. In some places, secondary cracks also formed parallel to the (010) surface from within the main microcracks, with further branching taking place deeper in the crystal. The cracks from the (010) surface extended to a depth of around 100 nm before radiating in other directions, suggesting that preparing particles with diameters smaller than this may be one way of suppressing microcrack formation and increasing the cycle lifetime of LiFePO_4_ positive electrodes^6^.

To determine the spatial distribution of Li ions in the crystal after chemical delithiation, we carried out quantitative valence EELS analysis. The intensity of the strong peak between 3 and 8 eV in the case of FePO_4_ (Supplementary Fig. 2) is attributable to interband transitions from states at the top of the valence band, and is closely related to the local valence state of Fe ions^29,36^. Fe valence states can be related to the Li content according to the formula ${\mathrm{Li}}_{1 - x}{\mathrm{Fe}}_{1 - x}^{2+ }{\mathrm{Fe}}_x^{3+ }{\mathrm{PO}}_4$, so measuring the intensity of the strong peak allows the local Li concentration to be estimated (Supplementary Fig. 3). Details of this method have been reported elsewhere^30^, and a brief summary is provided in Supplementary Note 2.

Figure 1d shows an Li concentration map of the area in Fig. 1c. Dark blue corresponds to Li-rich regions (*x* < 0.2) and orange–yellow to delithiated regions (*x* ≈ 1). The brightest region (with *x* > 1.1 according to the intensity scale bar) is amorphous carbon, which exhibits a strong peak corresponding to the *π* plasmon resonance (Supplementary Fig. 2b). The boundary between FePO_4_ (*x* ≈ 1) and Li_1-*x*_FePO_4_ (*x* < 0.2) phases is readily distinguishable, with contrast levels between those of the Li-rich and Li-poor phases. The pseudo-orthorhombic lattice parameters for the boundary region are also intermediate between those of the end-member phases, as confirmed by electron diffraction (see Supplementary Note 3 and Supplementary Fig. 4).

A magnified view of the top-right region of Fig. 1d is shown in Fig. 1e. This reveals that the interface between the Li-poor phase and boundary or interphase layer is demarcated by more-or-less well-defined crystal layers parallel to (100)_o_ and (210)_o_ planes, as seen in Fig. 1e, where subscript ‘o’ refers to the orthorhombic structure. Boundaries parallel to (310)_o_, (110)_o_ and (010)_o_ were also observed in some places (Supplementary Fig. 5). In contrast, there are no well-defined interfaces between the boundary layer and Li-rich phase. The interface between the lithiated phase and boundary layer thus comprises a gradual transition in Li content, whereas the interface between delithiated phase and boundary layer corresponds to a more abrupt or discrete change in Li content.

### Monoclinic subphase within the boundary layer

**Fig. 2:**
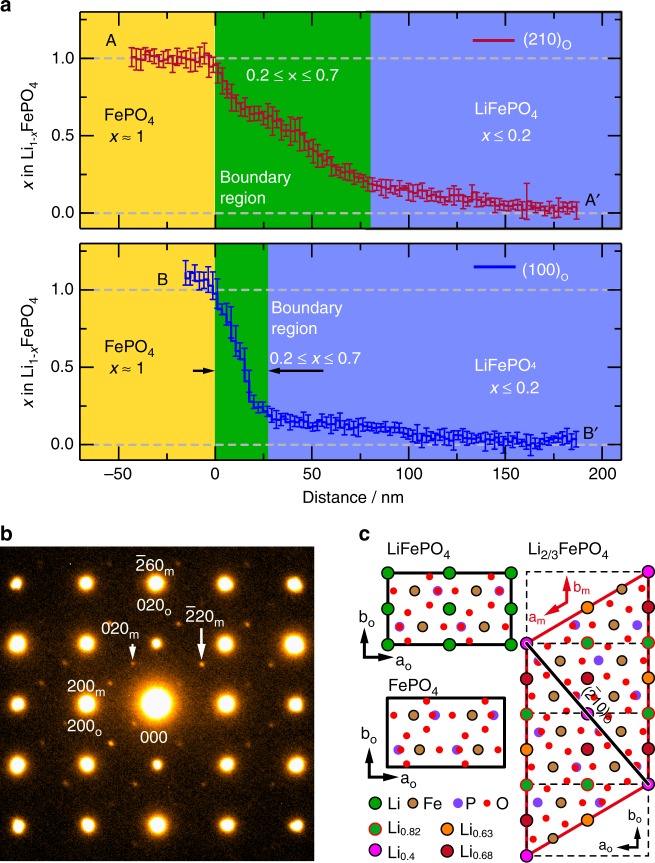
Boundary region between LiFePO_4_ and FePO_4_ after chemical delithiation. **a** Li concentration profiles along cross sections bounded by white dashed boxes in Fig. 1d. **b** Electron diffraction pattern obtained from the circled region in Fig. 1d. **c** Crystal models of Li_1-*x*_FePO_4_ for *x* ≈ 0, 1 and 0.34 (the model for *x* ≈ 0.34 is based on that reported in ref. ^11^). Subscripts “o” and “m” refer to orthorhombic and monoclinic structures, respectively

Figure 2a shows Li concentration profiles obtained by averaging seven line scans across the regions enclosed by elongated, dashed boxes in Fig. 1d, one oriented perpendicular to the direction of facile Li-ion diffusion (i.e., perpendicular to (100)_o_) and the other parallel to it (i.e., perpendicular to (010)_o_), with the ends inclined parallel to a (210)_o_ segment of the interface. Error bars show the standard deviations. The Li vacancy concentration perpendicular to (010)_o_ drops most steeply from *x* ≈ 1 to *x* ≈ 0.7 across the FePO_4_/interphase interface, and then gradually decreases across the boundary layer up to the interface with the Li-rich phase. The width of the boundary layer, with Li concentrations between 0.7 and 0.2, is about 70 nm at this location. The width in the [100]_o_ direction is considerably narrower, at about 20 nm. The boundary width thus appears to vary with facet orientation, most likely because of anisotropy in the lattice mismatch strains. For example, lattice mismatch between the delithiated and lithiated phases along [100]_o_ is about 3.6%, compared with 5.0% along [010]_o_ and 4.7% along [210]_o_. The differences in boundary width can thus be rationalised in terms of lattice mismatch; the larger the mismatch, the thicker the boundary region needed to reduce lattice strain between the two end-member phases.

The electron diffraction pattern in Fig. 2b was obtained from the region spanning the Li-rich/Li-poor boundary enclosed by the white circle in Fig. 1d. Extra spots (indicated by white arrows) not present in the patterns of FePO_4_ or LiFePO_4_ appear, corresponding to a monoclinic structure (Supplementary Fig. 6). One candidate phase for the intermediate layer is Li_2/3_FePO_4_, belonging to monoclinic space group *P*2_1_/*n*, as first reported by Boucher et al.^10^ and examined in more detail by Nishimura et al.^11^. The structure contains Li-poor and Li-rich atom columns ordered in the manner shown in Fig. 2c.

**Fig. 3:**
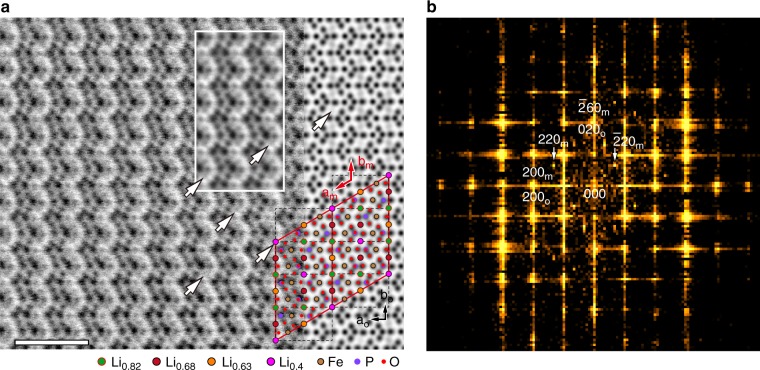
Direct observation of a monoclinic phase with Li vacancy ordering. **a** ABF STEM image of the monoclinic phase identified by electron diffraction taken down the [001]_o_ zone axis (left) compared with a simulated image calculated using the monoclinic unit cell of Li_2/3_FePO_4_ (right). Scale bar, 1 nm. The white rectangle shows an integrated ABF STEM image generated by summing intensities from different areas of the monoclinic phase. White arrows indicate positions of Li-poor columns corresponding to Li sites of occupancy 0.4 in the (overlaid) model of monoclinic Li_2/3_FePO_4_. **b** Two-dimensional Fourier transform of the left-hand image in **a**. *hkl*_o_ and *hkl*_*m*_ denote indices of spectral frequencies for simple orthorhombic and monoclinic unit cells, respectively

Figure 3a shows an atomic-scale image of the monoclinic subphase within the boundary layer obtained in annular bright-field (ABF) mode together with a simulated ABF image of the monoclinic Li_2/3_FePO_4_ structure. Dark spots in both images correspond to atom columns. The advantage of ABF imaging over high angle annular dark-field (HAADF) imaging is that the former enables atom columns of light elements such as oxygen and lithium to be observed simultaneously with those of heavier elements^21,22,27^. When vacancies are present in particular columns, the columns become brighter than those of columns with fully occupied sites^27^, and the greater the brightness the greater the concentration of vacancies. In Fig. 3a, there is only a slight difference in contrast between Li-poor and Li-rich columns in the experimental and simulated images, consistent with their site occupancies only being moderately different (from 0.4 to 0.8).

A two-dimensional Fourier transform of the experimental ABF image in Fig. 3a is shown in Fig. 3b. Spectral frequencies related to a monoclinic phase, specifically 220_m_ and $\bar 220_{\mathrm{m}}$, are clearly visible. The inset in Fig. 3a shows an ABF-STEM image produced by integrating the intensities of different areas of the boundary region, each the size of a monoclinic unit cell. Line profiles of the inset image reveal an ordering of Li-poor columns (Supplementary Note 4 and  Supplementary Fig. 7) in agreement with the profile calculated using the monoclinic structure model in Fig. 2c. The results of the Fourier transform and the average intensity profile thus confirm that the boundary region contains a monoclinic subphase with composition close to Li_2/3_FePO_4_. The lattice coherency, compositional gradient and absence of further additional spots in the Fourier-transformed images suggest that the rest of the boundary region consists of olivine-structured crystal with orthorhombic symmetry and randomly distributed Li vacancies.

**Fig. 4:**
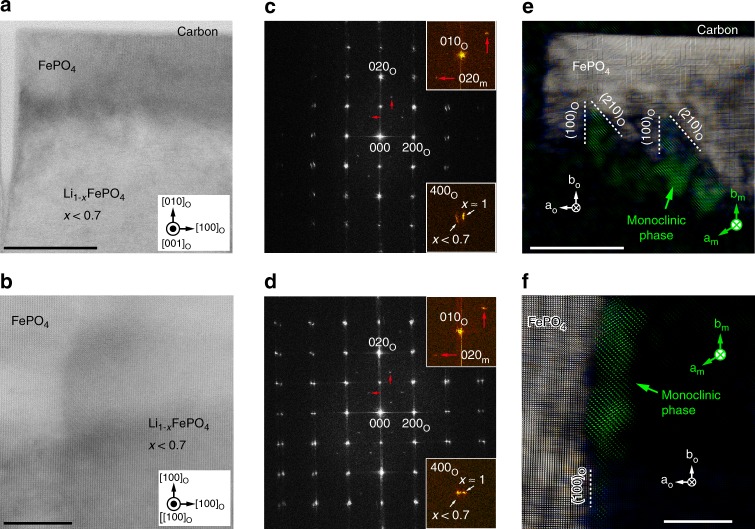
Distribution of the monoclinic phase in the boundary layer. **a**, **b** A BF STEM images of the region in Fig. 1c. and a boundary layer parallel to (100)_o_, respectively. Scale bars, 50 and 20 nm, respectively. **c**, **d** Two-dimensional Fourier transforms of the images in **a**, **b**, respectively. **e** A composite image comprising images of the Li-poor (Supplementary Fig. 8d) and monoclinic (Supplementary Fig. 8f) phases in **a** showing the local distribution of the monoclinic phase in the boundary near a vertical crack. Scale bar, 50 nm. **f** A composite image comprising images of the Li-poor and monoclinic phases in **b** prepared in the same manner as **e**. Scale bar, 20 nm. *hkl*_o_ and *hkl*_*m*_ denote indices of spectral frequencies for simple orthorhombic and monoclinic unit cells, respectively

To examine the distribution of the monoclinic subphase within the boundary region, data were extracted from the BF STEM images of Fig. 4a, b using a two-dimensional Fourier transform technique^37^. Figure 4c, d shows two-dimensional Fourier transforms of Fig. 4a, b, respectively. Spectral frequencies related to Li-poor (*x* ≈ 1), Li-rich (*x* < 0.7) and monoclinic phases are readily distinguished, making it possible to extract data from each phase separately. Details are provided in Supplementary Note 5 and Supplementary Figs. 8 and Fig. 9 . Figure 4e, f shows composite images of Li-poor and monoclinic phase images extracted from the Fourier transforms in 4c and 4d, respectively. The monoclinic phase can be seen to form closer to the FePO_4_ phase, with well-aligned facets (Fig. 4e, f), than to the LiFePO_4_ phase, even though its Li content is relatively high (0.67). In the non-monoclinic regions of the boundary layer, the Li content was found to vary between *x* ≈ 0.3 and *x* ≈ 0.7, with no noticeable ordering of Li vacancies.

To investigate the monoclinic subphase in more detail, we performed density functional theory calculations using a simplified model of experimentally observed Li_2/3_FePO_4_ (Supplementary Fig. 10). Details of the model are provided in Supplementary Note 6. The band gap of monoclinic Li_2/3_FePO_4_ was calculated to be 1.0 eV, considerably less than that of LiFePO_4_ at 3.6 eV and FePO_4_ at 1.9 eV, but consistent with that calculated by Ong et al.^38^ for an isolated hole polaron in LiFePO_4_. Plots of the partial densities of states reveal that the narrow band gap is a result of the high concentration of hole polarons associated with Fe^3+^ ions forming a low-energy conduction band (Supplementary Figs. 11 and 12). As electronic conduction within LiFePO_4_/FePO_4_ is known to occur by polaron hopping^9,38^, this can explain the higher electronic conductivities reported for the solid-solution (metastable) phase. During fast charging/discharging or under far-from-equilibrium conditions, the volume of the intermediate phase can also extend to the surface of the particle^39^, and its higher electronic conductivity (relative to the two end-member phases) is thought to play an important role in facilitating charge transfer during Li intercalation and deintercalation.

### Migration of the FePO_4_/LiFePO_4_ phase boundary during crystal relaxation

After partial delithiation, the biphase interface is not static but exhibits gradual structural relaxation as a result of reverse Li-ion migration from the crystal bulk back to the surface. This occurs topotactically because the boundary region enables the crystal lattices of the two main phases to remain connected coherently and thus the [010]_o_ Li-ion channels remain intact. As this process proceeds, the crystal expands back to its original volume, reducing the volume of the Li-poor phase and eventually closing cracks that formed at the delithiated surface.

**Fig. 5:**
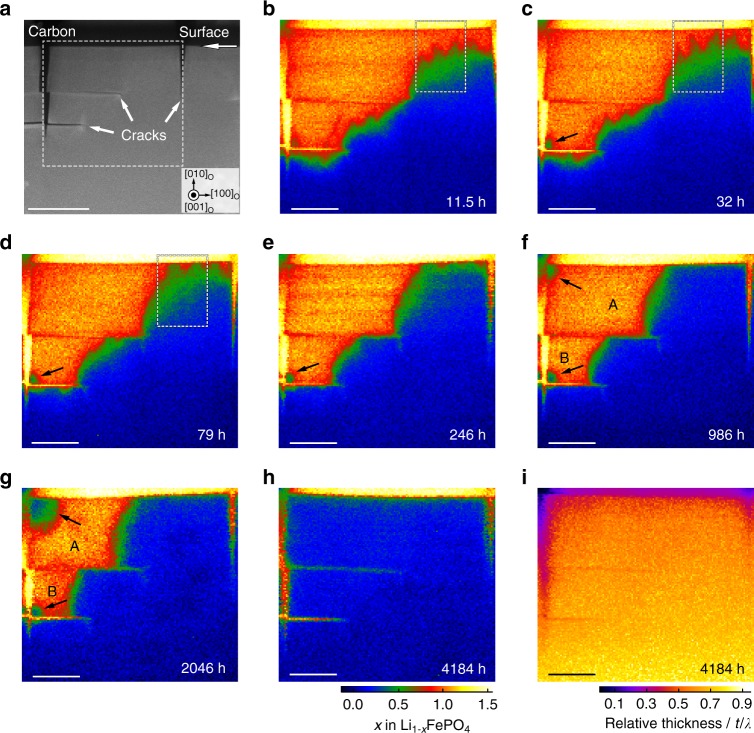
Migration of the FePO_4_/LiFePO_4_ phase boundary. **a** ADF STEM image after delithiation showing cracks perpendicular and parallel to the (010) surface. Scale bar, 200 nm. **b**–**h** Li concentration maps of the region bounded by the white dashed rectangle in **a** obtained (**b**) 11.5 h, (**c**) 32 h, (**d**) 79 h, (**e**) 246 h, (**f**) 986 h, (**g**) 2046 h and (**h**) 4184 h after delithiation. Scale bar, 100 nm. The colour scale bar below **h** shows colour changes as a function of *x* for maps **b**–**h**. Black arrows in **c**–**g** indicate corner regions to which Li ions returned by external diffusion to form a separate region of intermediate Li content. **i** Map of relative thickness *t*/*λ* taken at the same time as **h**. Scale bar, 100 nm. The colour scale bar below **i** shows colour changes as a function of *t*/*λ*

The observed changes taking place at the biphase interface during this relaxation/healing process provide insights into the mechanism by which Li migrates between the Li-rich and Li-poor phases. Figure 5a shows an ADF STEM image of a region spanning the boundary region soon after Li removal. Li concentrations were also mapped from the same region at several different times between 11.5 h and >4000 h after oxidation, and the results are shown in Fig. 5b–h. (The same results plotted as Li concentration gradation maps are provided as Supplementary Fig. 13.) Figure 5i shows a map of relative thickness *t/λ* for the area in Fig. 5h. As the mean free path, *λ*, of stoichiometric LiFePO_4_ is 106.3 nm, the map shows that the thickness of the relaxed structure varied from around 50 nm near the surface to 80 nm inside the crystal.

Between 11.5 and 79 h (Fig. 5b–d), the Li ions can be seen to have migrated in the [010] direction from the LiFePO_4_ crystal bulk back to FePO_4_ surface regions. In some places, lateral cracks parallel to the (010) surface physically separated delithiated regions from the crystal bulk in the [010] direction, as seen in Fig. 5e. In these cases, Li ions were unable to migrate back to the separated areas along the main transport paths, viz., [010] channels.

Between 246 and 2046 h after delithiation (Fig. 5e–g), Li ions can also be seen to have migrated progressively from the LiFePO_4_ region to the FePO_4_ regions in the [100]_o_ direction. As the overall interface becomes aligned with the (100)_o_ plane, the width of the intermediate layer decreases, as does the width of region **A**, most readily seen in Fig. 5f, g. Interestingly, of the two main FePO_4_ regions, **A** decreases in size more rapidly than **B**. This may be because the larger region (**A**) produces a larger strain field, providing a larger driving force for Li to relax back into the delithiated region. As seen in Fig. 5h, after a long period of time (over 5 months), both regions had almost completely re-lithiated, despite being separated from the crystal bulk in the [010] direction. According to the gradation map in Supplementary Fig. 13h, the residual Li vacancy concentration at the surface was between 10 and 30%.

In addition to relaxation of Li back into the delithiated regions from the bulk crystal in [010] and [100] directions, Fig. 5f, g reveal that intermediate phases also formed at corners of some cracks (e.g., regions indicated by black arrows in Fig. 5g). These regions also contained the monoclinic phase (Supplementary Note 7 and Supplementary Fig. 14). As electron beam damage and sample thickness effects can be discounted as the cause of this phenomenon (more details are provided in Supplementary Note 7 and Supplementary Fig. 15), it appears that Li ions have migrated around the surfaces and re-entered the crystal at the edges, which are regions of high stress concentration.

**Fig. 6:**
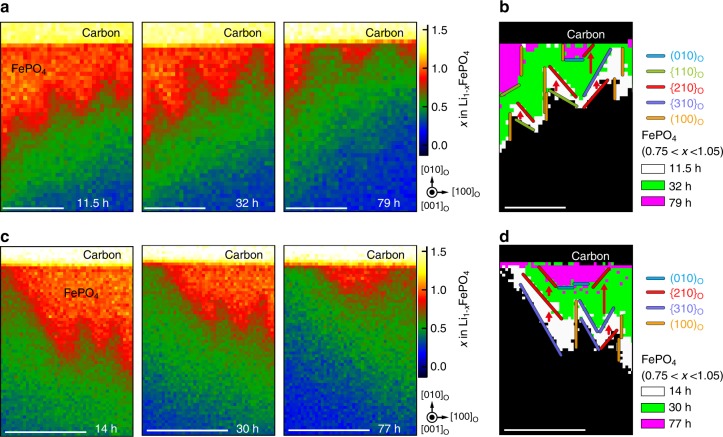
Changes in boundary planes between FePO_4_ and the intermediate region during relaxation. **a** Magnified view of the Li concentration map of the region enclosed by the dotted rectangles in Fig. 5b–d (from left to right) 11.5, 32 and 79 h after delithiation. **b** Superimposed binarised images of the region in **a** 11.5 h (white), 32 h (green) and 79 h (magenta) after delithiation. **c** Magnified view of the Li concentration map of the region enclosed by the dotted rectangle in Supplementary Fig. 4 (from left to right) 14, 30 and 77 h after delithiation. **d** Superimposed binarised images of the region in **c** 14 h (white), 30 h (green) and 77 h (magenta) after delithiation. In the binarised images, “FePO_4_” was taken to correspond to Li vacancy concentrations between 0.75 and 1.05. Scale bar, 50 nm

The Li concentration maps also reveal that the orientations of facet planes between the boundary region and delithiated phase vary as a function of time; although, the interface retains a jagged morphology whenever it is present. Figure 6a shows magnified views of Li concentration maps of the region within the dotted rectangles in Fig. 5 11.5 h, 32 h and 79 h after delithiation. Superimposed binarised images in Fig. 6b divided into “FePO_4_” (Li concentrations between 0.0 and 0.25 moles per formula unit) and “non-FePO_4_” (Li concentrations greater than 0.75 moles per formula unit) regions show how the area of the FePO_4_ phase decreases with increasing relaxation time. Figure 6a, b show that facets of the FePO_4_/boundary layer interface after 11.5 h are parallel to (110)_o_, ($\bar 210$)_o_ and (100)_o_ planes. As relaxation continued, a succession of changes in facet orientation occurred, as indicated by red arrows, with many (110)_o_ oriented interfaces changing to ($\bar 210$)_o_, and ($\bar 210$)_o_ interfaces changing first to ($\bar 310$)_o_ and then back to ($\bar 210$)_o_, accompanied by formation of narrow (010)_o_ facets. Facets at other portions of the boundary layer after 14 h were parallel to ($\bar 210$)_o_, (310)_o_ and (100)_o_ planes, as seen in Fig. 6c, d. In this case, (310)_o_ interfaces changed to (210)_o_, and ($\bar 210$)_o_ to ($\bar 310$)_o_ and then back to ($\bar 210$)_o_ as time proceeded. In other words, as structural relaxation progressed, many boundary facets tended to align with (*h*10)_o_ planes with higher *h* values, eventually becoming parallel to planes with *h* → ∞, i.e., {100}_o_ planes. At the same time, Fig. 6 shows that some higher index facets changed to lower index facets, including some parallel to {010}_o_, i.e., with *h* ≈ 0. The {010}_o_ oriented facets were observed to occur in regions where the size of the FePO_4_ region was small, suggesting that low-index planes such as {010}_o_ are stable in smaller strain fields. Although beyond the scope of this work, to elucidate completely the dynamics of changes from high-index to low-index facets, the effect of both Li-ion migration rates and the complex misfit strain fields surrounding the jagged interface morphology need to be examined in detail.

Observation of changes in the crystal morphology and local composition also provided insights into the relaxation process after partial delithiation on a larger scale. The main microcracks formed mainly parallel to the (100) plane (see Supplementary Figs. 1c and 1d), consistent with the misfit strain along [001]_o_ being the smallest of those along the principal axis directions. Calculation of a simplified lattice mismatch, *ε*_<*hkl>*_, defined as *ε*_<*hkl>*_ = (*d*_<*hkl>*_^R^ – *d*_<*hkl>*_^P^)/*d*_<*hkl>*_^R^ (where *d*_<*hkl>*_^R^ is the *d*-spacing along <*hkl*>directions of the Li-rich phase, and *d*_<*hkl>*_^P^ is the *d*-spacing along <*hkl*>directions of the Li-poor phase), gives values of *ε*_**<001>**_ for interfaces LiFePO_4_/FePO_4_ and Li_2/3_FePO_4_/FePO_4_ of −1.9% and −1.0%, respectively^11,14^. These misfit values are consistent with the overall trends, but a detailed explanation of the observed interfaces between the three phases requires closer analysis of the effect of crystal anisotropy on misfit strain.

Pseudo-orthorhombic lattice parameters of the Li_2/3_FePO_4_ phase have been reported to be *a*_o _= 0.5935 nm and *b*_o _= 1.0202 nm^11^. Using these values, misfit strains *ε*_***<*010*>***_, *ε*_***<*110*>***_, *ε*_***<*210*>***_, *ε*_***<*310*>***_ and *ε*_***<*100*>***_ between Li_2/3_FePO_4_ and FePO_4_ (*a*_o_ = 0.5789 nm and *b*_o_ = 0.9814 nm) are calculated to be 3.80, 3.75, 3.25, 3.08 and 2.47%, respectively, showing that higher index lattice planes have smaller lattice mismatch. Values of *ε*_<*hkl>*_ between LiFePO_4_ and Li_2/3_FePO_4_ along the same directions are all <2%, suggesting that a small lattice mismatch results in a diffuse interface, in this case perpendicular to [010], as seen in Fig. 1. The observation of diffuse interfaces is in good agreement with a recent synchrotron microbeam diffraction study^20^. In contrast, the large lattice mismatch in the [010] direction between FePO_4_ and Li_2/3_FePO_4_, coupled with this being the fastest direction for Li-ion migration, produces sharp, well-defined boundaries on the FePO_4_ side of the boundary layer. The range of Li concentrations across the boundary region between the Li-poor and Li-rich phases suggests that local misfit strain is indeed able to alter the relative stabilities of Li_*x*_FePO_4_ phases, as predicted by simulation^17^.

## Discussion

Our STEM observations and EELS measurements reveal how the delithiated surface region of an LiFePO_4_ crystal relaxes as a function of time to return to an essentially monophasic state when a comparatively small amount of Li has been removed. The driving force for Li-ion migration into delithiated regions in this case is the large chemical potential difference between the two main phases, together with the lattice strain field that exists across the boundary layer. Further details are provided in Suapplementary Note 8 and Supplementary Figs. 16 and 17. The strain field has been reported to extend to about 10–25 nm^40^, consistent with the more-or-less constant width of the boundary region during the relaxation process until it disappears upon reaching the crystal surface.

The room-temperature self-diffusion coefficient of Li in FePO_4_ in the [010] direction was estimated directly from the rate of shrinkage of the delithiated regions in the binarised images in Fig. 6 to be 4.1 × 10^−17^ cm^2^ s^−1^. Similarly, the diffusion coefficient in the [100] direction was estimated from regions **A** and **B** in Fig. 5 to be 3.2 × 10^−18^ cm^2^ s^−1^ and 9.2 × 10^−19^ cm^2^ s^−1^, respectively, indicating that the magnitude of the Li diffusion coefficient is related to the strain field, and that Li diffusion in the [010] direction is at least an order of magnitude faster than in the [100] direction^31,32^. Previous experimental studies^41–45^ reported Li diffusion coefficients between 10^−17^ and 10^−^^9^ cm^2^ s^−1^. The wide scatter in these values is not unexpected given the different measurement methods used. In particular, concentrations of antisite defects, which are known to hamper Li diffusion^23,46^, depend on the method and conditions used during synthesis. Although our estimates of the diffusion coefficient have large uncertainties, as far as we are aware this is the first time that such calculations have been made by directly measuring the migration of the biphasic boundary with time.

Our observations of chemically delithiated (010) surfaces of LiFePO_4_ are consistent with a Li-poor shell/Li-rich core model, in excellent agreement with earlier electron microscopy^25^ and microbeam X-ray diffraction studies^20^. In particular, our observation of a narrower boundary layer in the [100] direction compared to [210] and higher index directions by estimating the Li content is consistent with the trend reported by Nakamura et al. based on changes in lattice spacings^25^. In contrast to their work, the volume of lithium removed from the crystal in our case was much smaller than that remaining in the crystal, and thus a large chemical potential difference existed across the biphasic boundary layer after delithiation. It is not clear what volume fraction of Li needs to be removed before the Li-poor phase can coexist in equilibrium with the Li-rich phase, but the large chemical potential difference in our case provided sufficient driving force for Li atoms from the Li-rich bulk to re-lithiate the Li-poor surface by diffusion through the boundary layer. This resulted in the simultaneous motion of the boundary layer to the surfaces (both the original (010) surface and those exposed by cracking) in the manner described in the previous section and reduction in the volume of Li-poor phase, with no noticeable change in the Li content of the crystal bulk.

Wagemaker and coworkers^20^ showed that the width and sharpness of the boundary layer vary depending on the delithiation rate, with more diffuse boundaries, which appear to be favourable for fast charge/discharge kinetics and electrode lifetime, forming under higher cycling rates. Our STEM and EELS results show that at the atomic level the interface has its own distinct morphology, with an inherently diffuse LiFePO_4_/boundary layer interface and a facetted FePO_4_/boundary layer interface whose local orientations shift and realign as re-lithiation of the Li-poor surface layer proceeds. Consistent with earlier theoretical results^31,32^, Li-ion migration was seen to proceed most rapidly in the [010] direction, with much slower migration in the [100] direction. This further emphasises the importance of the (010) surface for enabling rapid charging and discharging of olivine-type cathode materials.

In our case, chemical delithiation would have rapidly removed Li atoms from the (010) surface, so it is likely that at first an extended solid-solution region was formed, such as has been reported by Hess et al. from combined operando XRD-electrochemical impedance spectroscopy measurements^47^. Using first-principles calculations, Abdellahi et al.^17^ showed that spinodal decomposition of an intermediate solid-solution phase preferentially leads to the formation of an *ac*-oriented boundary rather than the elastically favoured *bc*–oriented boundary that is often observed in electrochemically delithiated particles. The biphasic interfaces observed in this study are thus likely to resemble those in LiFePO_4_ particles subjected to rapid charging. Our observation of multiphase/multidomain boundary regions is also consistent with Hess et al.’s results for high-rate electrochemically delithiated crystals^47^. This may explain why the FePO_4_/boundary region observed in our study is more facetted than that predicted from first-principles studies of the Li_2/3_FePO_4_ structure by Boucher et al.^10^, which corresponds to a *bc*-oriented LiFePO_4_/FePO_4_ boundary in a slowly delithiated crystal. Formation of *ac*-oriented boundaries in rapidly charged particles should also favour rapid discharging because a greater area of delithiated phase is normal to the preferred Li-ion intercalation direction. Depending on the discharge rate, interfaces between delithiated and re-lithiating phases may be either facetted or diffuse during intercalation of Li ions, but for rapidly charged and discharged crystals biphasic boundaries are expected to resemble the structures observed in this study after relaxation.

Quantitative nano-level EELS measurements also confirmed earlier inferences that the Li content varies gradually across the boundary phase. However, although the average Li content was found to vary more-or-less linearly from one side of the biphase boundary to the other, at the atomic level our observations revealed a more complex situation in which the boundary region consists not of a single phase, as had been assumed previously, but of a mixture of two or more phases with slightly different crystal symmetries, Li contents, Li-vacancy ordering and lattice strains. Interestingly these subphases remain coherently bonded to one another, with no dislocations observed in either the Li-rich, Li-poor or interface regions, and only the monoclinic subphase exhibited a crystal symmetry different to that of the end-member phases. Given the complexity and metastable nature of the boundary layer, the size, distribution and Li contents of these subphases likely vary as a function of time and degree or rate of (de)lithiation.

The above results help explain how the interface layer alleviates lattice strain between the two thermodynamically stable end-member phases while retaining a high degree of structural integrity. These insights have implications for the development not only of olivine-type cathode materials but more generally for other topotactic materials in which non-equilibrium solid-solution transformation mechanisms can be exploited to achieve improved rate capabilities.

## Methods

### Experimental procedure

A commercially available LiFePO_4_ single crystal (Oxide Corp.) was used for all experiments. The crystal was cut perpendicular to each principal axis and polished. The size of the single crystal was around 0.5 × 0.5 × 0.5 mm^3^. Samples with fresh fracture surfaces were obtained by manually applying a bending force to the crystal with a pair of tweezers (Supplementary Fig. 1a). Samples were delithiated by chemical oxidation in an acetonitrile solvent using NO_2_BF_4_ as the oxidant according to LiFePO_4_ + *x*NO_2_BF_4_ → Li_1-*x*_FePO_4_ + *x*LiBF_4_ + *x*NO_2_. The molar ratio *x* was about 0.2. After a reaction time of 3–5 min, the crystal was washed in acetonitrile several times to halt delithiation.

(010) surface samples before and after delithiation were coated with carbon (around 100 nm) for viewing by scanning electron microscopy (SEM). Cross-sectional scanning transmission electron microscopy (STEM) samples were prepared using a dual-beam focused ion beam (FIB) scanning microscope (NB5000, Hitachi High-Technologies Co.) with Ga ions. Damage layers were partially removed at 2 kV during the last stage of FIB, and completely removed by subsequent gentle Ar ion milling (PIPS, Gatan Inc.) using a cold stage at around −150 °C. The sample thickness varied from about 50 to 100 nm over the observed areas. To prevent contamination affecting the EELS spectra, hydrocarbon contaminants were removed from samples with an ion cleaner (JIC-410, JEOL Ltd.) before being placed in the microscope. The overall Li deficiency after delithiation was estimated to be around 0.2% (giving an overall composition of Li_≈0.998_FePO_4_) by comparing volumes of LiFePO_4_ and FePO_4_ regions in a freshly milled sample as described in Supplementary Note 9.

Between STEM experiments, samples were stored in two different ways. From about 8 to 80 h after delithiation, samples were kept in the electron microscope under a vacuum of ~8.0 × 10^–6^ Pa. After this time, samples were kept in a vacuum desiccator (around 150 Pa) at room temperature. Samples were transferred between FIB and microscope, and microscope and desiccator (and vice versa), in air with an exposure time of <10 min in each case.

The structure of the delithiated Li_1–*x*_FePO_4_ crystal was investigated by STEM using an aberration-corrected (CEOS GmbH) scanning transmission electron microscope (JEM-2100F, JEOL Ltd.). STEM observations were performed at accelerating voltages of 200 kV. The probe-forming aperture semiangle used was 17 mrad, and ABF STEM images were recorded with 10–23 mrad detectors. A radial difference filter (HREM Filters Lite v1.5.1, HREM Research Inc.) was applied to the image to reduce noise. ABF image simulations were generated using the program xHREM (HREM Research Inc.).

EELS spectra were obtained using an EELS spectrometer (Tridiem ERS, Gatan, Inc.) attached to a Wien-filter monochromated aberration-corrected STEM (JEM-2400FCS, JEOL Ltd.) operated at 200 kV. EELS spectra were recorded in STEM mode using 0.1 eV per channel and an energy resolution of 250–300 meV (full-width at half-maximum of zero-loss peak). The convergence and collection semiangles were 33 and 43 mrad, respectively.

### Computational procedure

The structure and stability of Li_2/3_FePO_4_ were investigated using first-principles calculations within the framework of density functional theory (DFT). The projected augmented-wave (PAW) method^48,49^ was used, as implemented in the VASP code^50–52^, with the following electron configurations treated explicitly in the pseudopotentials of neutral atoms: 2s^1^ for Li, 3p^6^ 3d^6^ 4s^2^ for Fe, 3s^2^ 3p^3^ for P and 2s^2^ 2p^4^ for O. The cutoff energy for planewave basis sets was 500 eV. The mesh size for ***k***-point sampling in the Brillouin zone was 2 × 3 × 4 for LiFePO_4_ and FePO_4_. For Li_2/3_FePO_4_, a ***k***-point sampling mesh of 2 × 2 × 4 was used. All calculations were carried out with the systems in a spin-polarised ferromagnetic state. The GGA + *U* approach^53^ was used to account for strong correlation effects of Fe *3d* orbitals with *U* set at 4.3 eV^54^, the mean value of parameters reported for Fe^2+^ and Fe^3+^ atoms by Zhou et al.^55^. Crystal structures were considered fully optimised once residual forces on all atoms were below 0.02 eV Å^–1^. Calculated lattice parameters were in good agreement with experimental values (Supplementary Table 1)

### Data availability

The data that support the findings of this study are available from the corresponding author upon reasonable request.

## Electronic supplementary material

Supplementary Information
