# Supplementary material for: Microscopic mechanism of biphasic interface relaxation in lithium iron phosphate after delithiation
Source: Nat Commun. 2018 Jul 20;9:2863. doi: 10.1038/s41467-018-05241-1 (PMC6054635; doi:10.1038/s41467-018-05241-1)
Supplement: Supplementary file 1 — Supplementary Information [file 41467_2018_5241_MOESM1_ESM.pdf]

Supplementary information

Microscopic mechanism of biphasic interface relaxation in lithium iron phosphate after delithiation

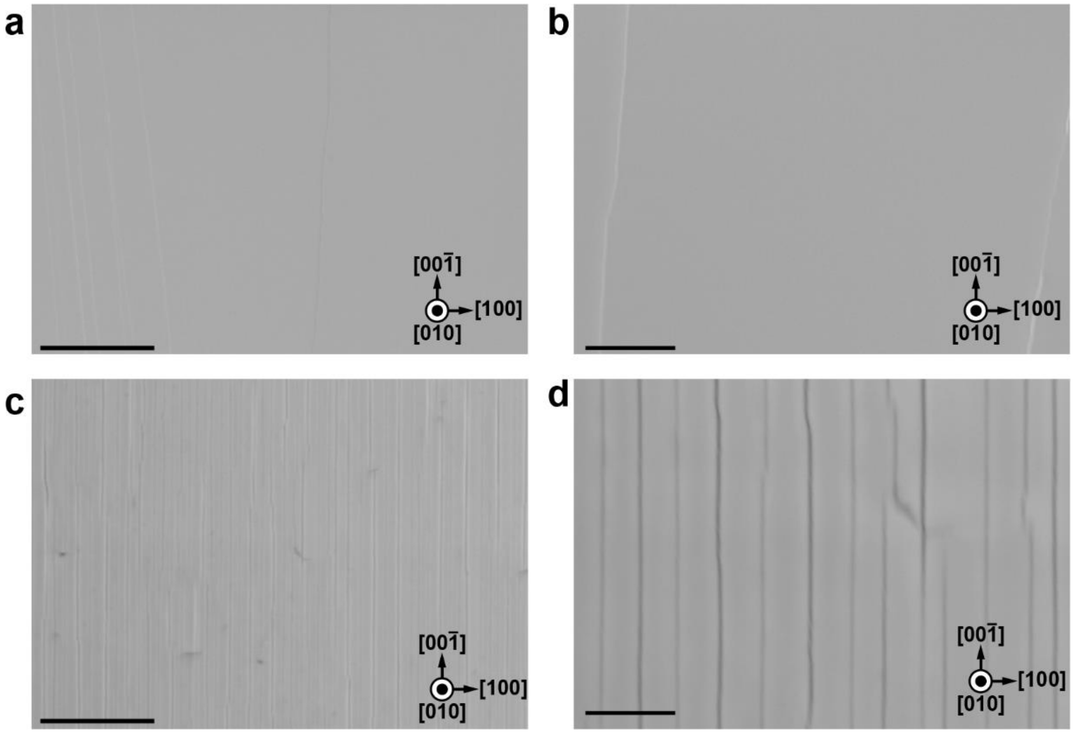

Supplementary Figure 1 | Surfaces before and after delithiation. a,**b**, SEM images of an as-cleaved surface. Scale bar, 5 µm. **c**,**d**, SEM images of a (010) surface after delithiation. Scale bar, 1 µm.

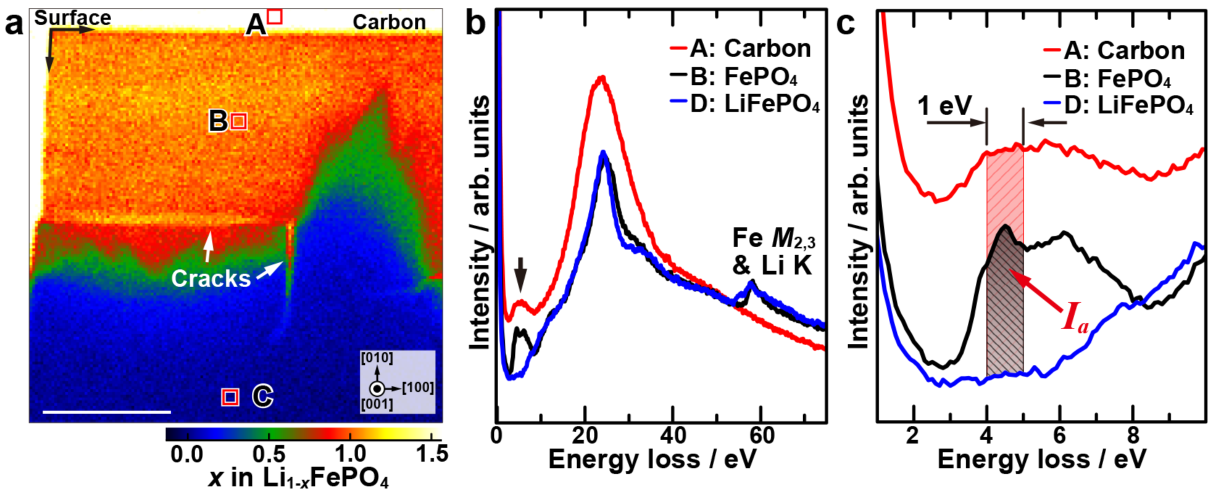

Supplementary Figure 2 | Valence EELS spectra of $Li_{1-x}FePO_4$ and amorphous carbon. **a**, Li concentration map generated from spectra of the single crystal after delithiation. Red squares A, B and C lie within regions of amorphous carbon, $FePO_4$ and $LiFePO_4$, respectively. Scale bar, 100 nm. **b**, Low-loss spectra of amorphous carbon**,** $FePO_4$ and $LiFePO_4$ extracted from mapping spectra from rectangles A, B and C in **a**. The black arrow indicates the interband transition peaks of the spectrum of $FePO_4$ and the π plasmon peak of amorphous carbon. **c**, Magnified view of **b** showing the 1.0-eV-wide integration region for the first interband transition peak of $FePO_4$ and the π plasmon peak of amorphous carbon.

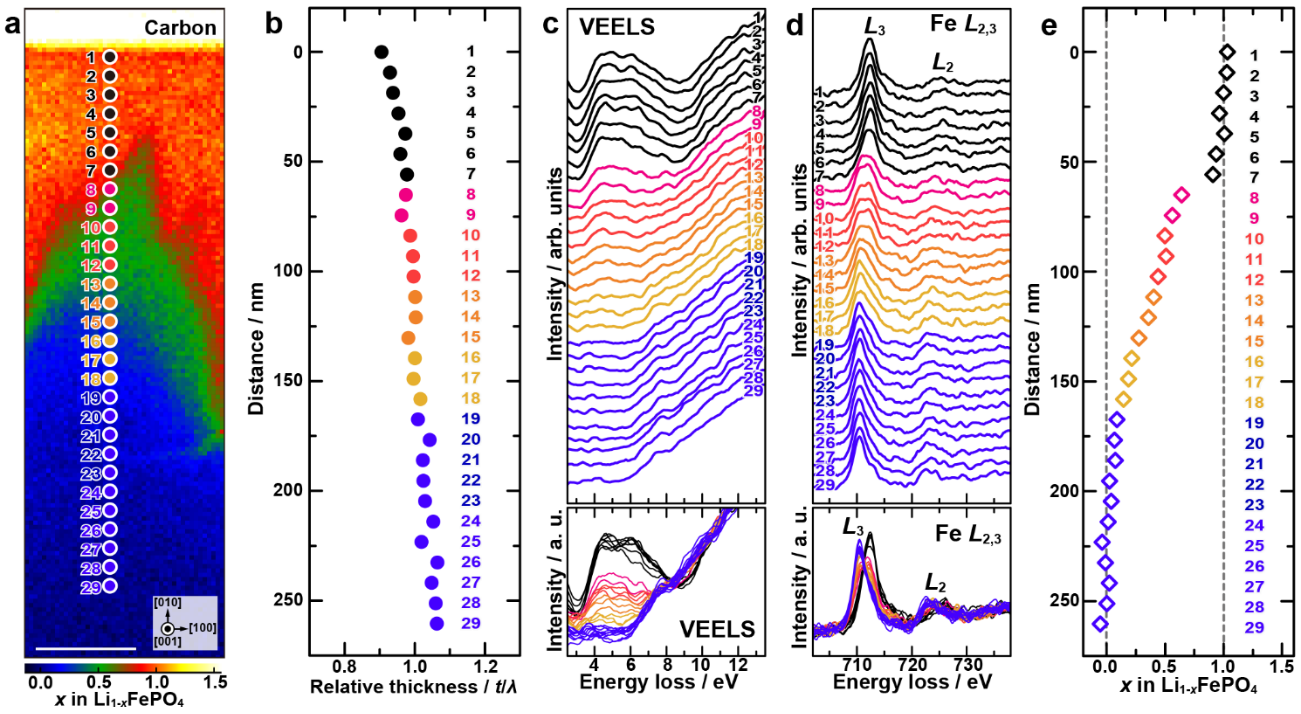

Supplementary Figure 3 | Li concentration mapping from EELS spectra. **a**, An Li concentration map of the region around an interface between $FePO_4$ and $LiFePO_4$, with line profile step positions numbered and colour coded (black: $FePO_4$; blue: $LiFePO_4$: others: intermediate). Scale bar, 50 nm. **b**, Relative sample thickness t/λ along the line profile. **c**, Valence EELS spectra obtained from numbered points in **a**. **d**, Fe $L_{2,3}$-edge spectra obtained from numbered points in **a**. Upper and lower plots in **c** and **d** show exploded views and normalised, overlaid views of the spectra, respectively. **e**, x in $Li_{1-x}FePO_4$ calculated from the line profiles in **c**.

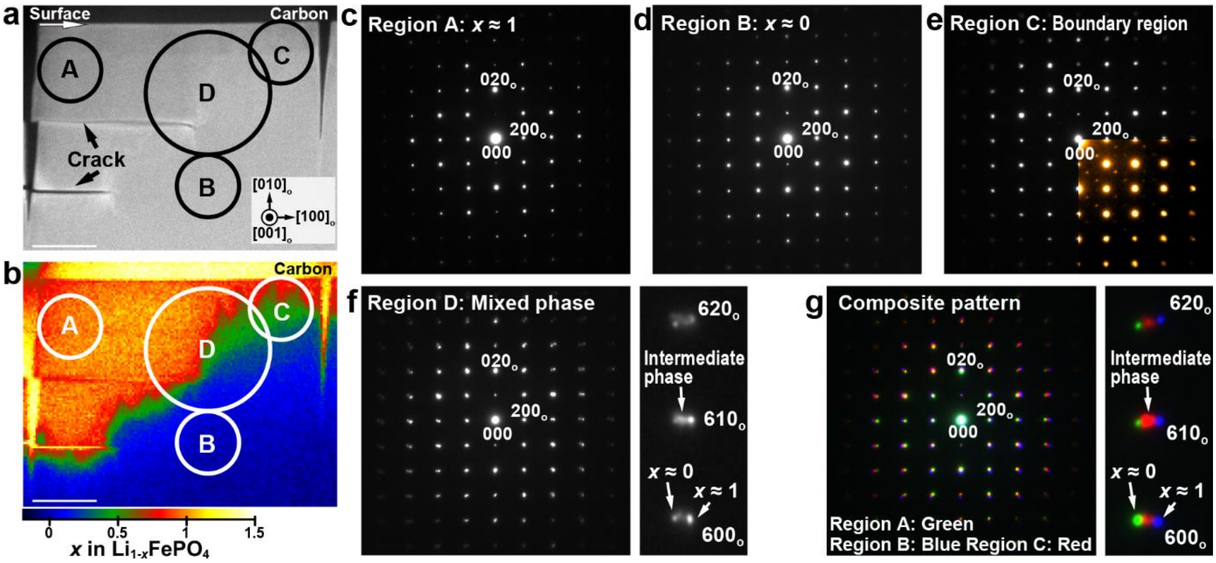

Supplementary Figure 4 | Relative lattice parameters from electron diffraction patterns. a, ADF STEM image of the crystal after delithiation. Scale bar, 100 nm. **b**, Li concentration map of the same crystal in **a**. Scale bar, 100 nm. Circles A, B and C in both **a** and **b** correspond to $FePO_4$, $LiFePO_4$ and boundary regions, respectively, while **D** spans all three regions. **c-f**, Electron diffraction patterns obtained from areas **A**, **B**, **C** and **D**, respectively, with a magnified view of 600, 610 and 620 spots shown on the right in the case of **f**. An enhanced colour diffraction pattern showing extra spots is overlaid in the case of the boundary region in **e**. **g**, Composite image of electron diffraction patterns in **c**, **d** and **e**, with a magnified view of 600, 610 and 620 spots shown on the right. Subscript “o” refers to the orthorhombic structure.

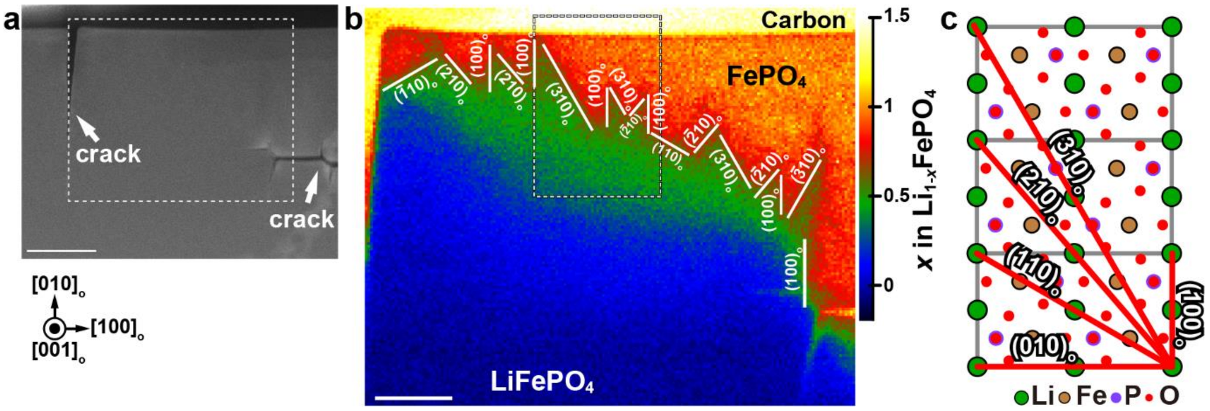

Supplementary Figure 5 | Boundary planes between $FePO_4$ and $Li_{1-x}FePO_4$. a, ADF STEM image of a (010) surface region of the single crystal after delithiation. Scale bar, 100 nm. **b,** Li concentration map of the region enclosed by the dashed rectangle in **a**. Scale bar, 50 nm. **c,** Schematic showing crystal planes in the $LiFePO_4$ structure when viewed down [001].

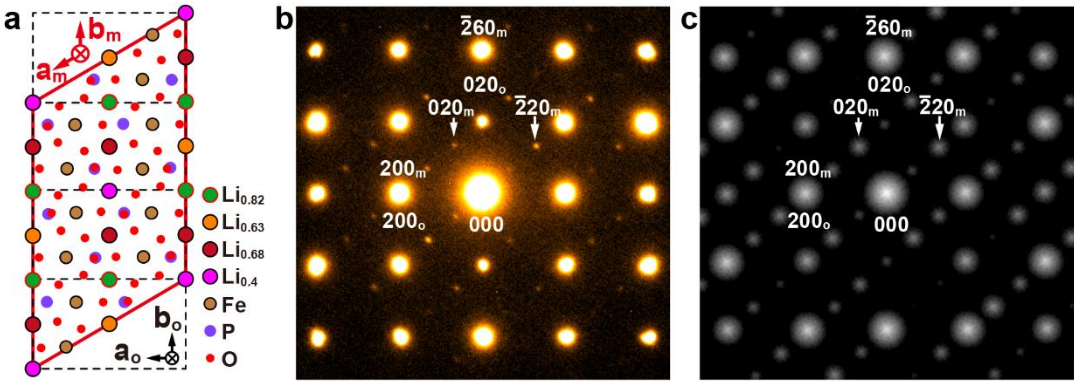

Supplementary Figure 6 | Experimental and simulated electron diffraction patterns of the monoclinic phase. a, Schematic of the monoclinic $Li_{2/3}FePO_4$ crystal model. The full red lines and dashed black lines indicate the monoclinic unit cell and orthorhombic unit cell, respectively. The orientation vectors at the top and bottom of the diagram are for the monoclinic ('m') and orthorhombic ('o') systems, respectively. **b,** Experimental electron diffraction pattern. **c,** Simulated electron diffraction pattern of the crystal model in **a**.

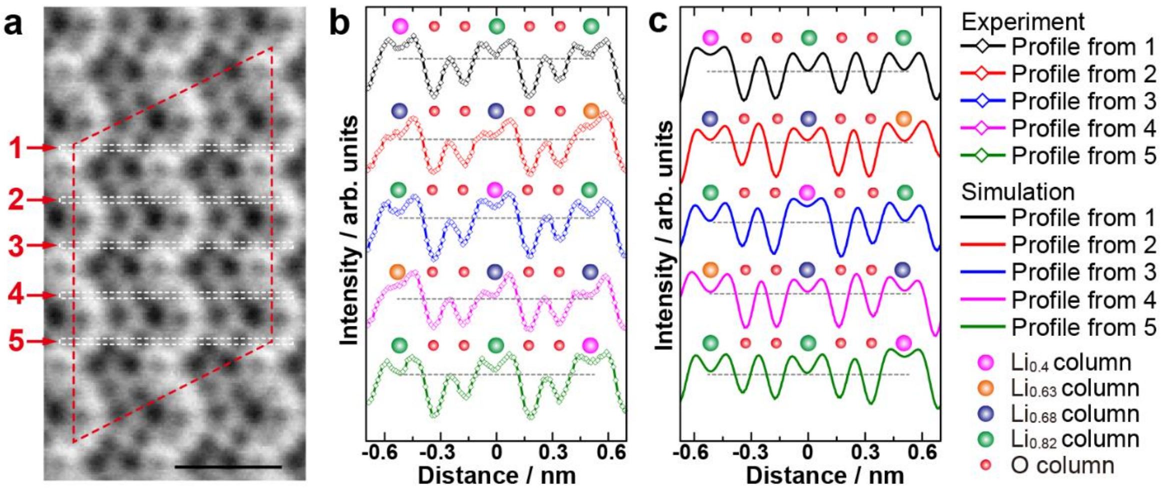

Supplementary Figure 7 | Line profiles from an ABF STEM image of the monoclinic phase. a, Integrated ABF STEM image of the monoclinic phase corresponding to the overlaid image in main text Fig. 3a. Red dashed lines demarcate a monoclinic unit cell. Scale bar, 0.5 nm. **b**, Intensity line profiles from regions 1-5 bounded by white dashed lines in **a**. **c**, Intensity line profiles from corresponding regions of the simulated ABF image generated using the structure for monoclinic $Li_{2/3}FePO_4$ reported in ref. 18. Dashed lines in **b** and **c** highlight intensity troughs corresponding to Li-rich columns.

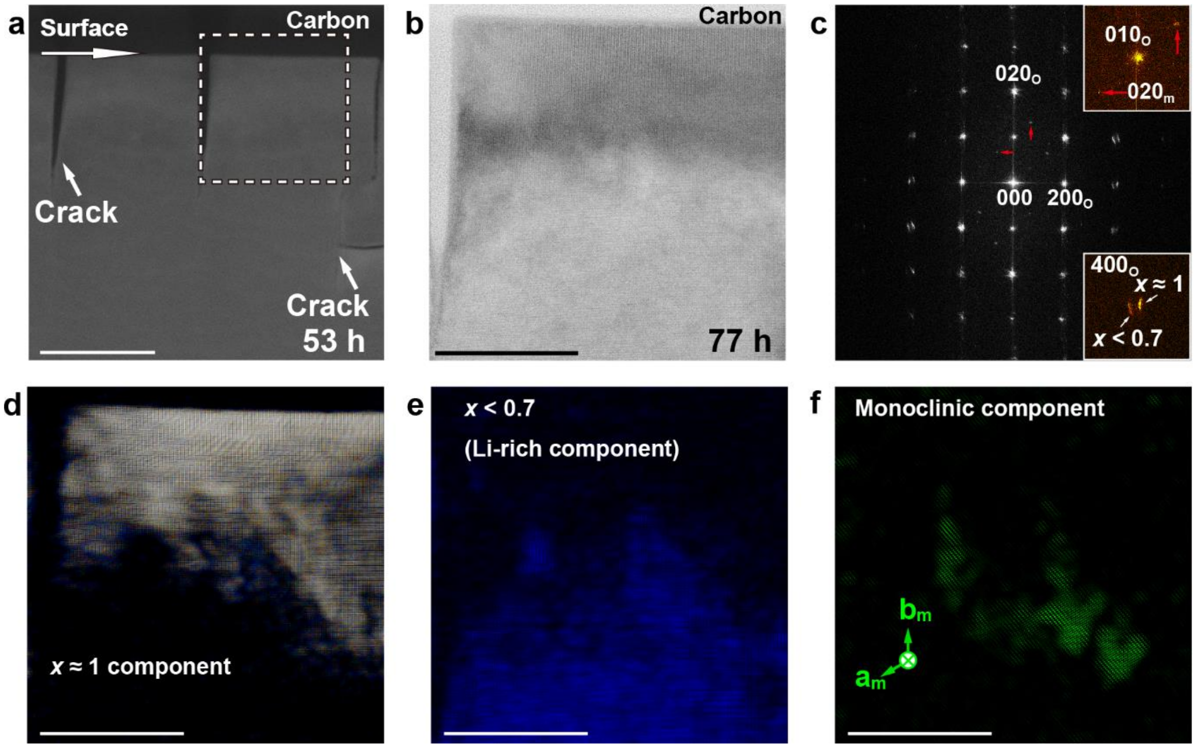

Supplementary Figure 8 | Extraction of monoclinic phase from BF STEM images. a, ADF STEM image of the crystal 53 h after delithiation (from main text Fig. 1c). Scale bar, 100 nm. **b**, ABF STEM image of the region enclosed by the dashed square in **a** 77 h after delithiation. Scale bar, 50 nm. **c**, Two-dimensional Fourier transform of the image in **b**. hkl_{o} and hkl_m denote indices of spectral frequencies for simple orthorhombic and monoclinic unit cells, respectively. **d-f**, Images constructed from inverse Fourier transforms of the group of (**d**) Li-poor ($x \approx 1$), (**e**) Li-rich ($x < 0.7$) and (**f**) monoclinic spectral frequencies in **c**, respectively. Scale bar, 50 nm.

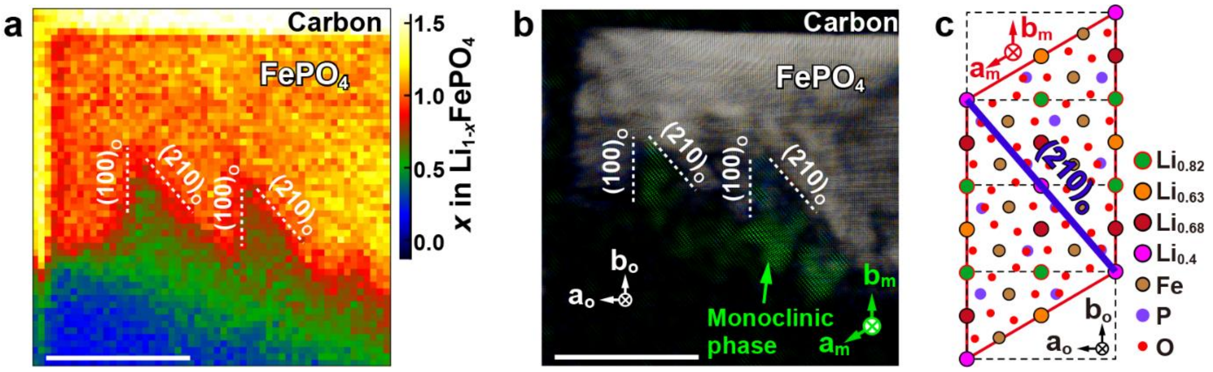

Supplementary Figure 9 | Distribution of the monoclinic phase in the boundary region of the delithiated crystal. a, Li concentration map of the same region as main text Fig. 1e. **b**, A composite image comprising extracted images of the Li-poor (Supplementary Figure 8d) and monoclinic (Supplementary Figure 8f) phases showing the distribution of the monoclinic phase. Scale bar, 50 nm. **c**, A schematic of the monoclinic $Li_{2/3}FePO_4$ crystal lattice oriented the same as the monoclinic phase in **b**. Full red lines and dashed black lines indicate monoclinic unit and orthorhombic unit cells, respectively.

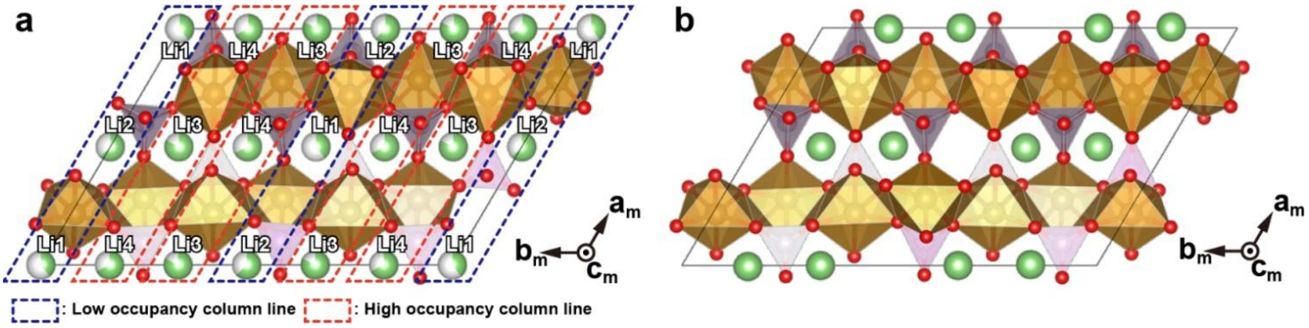

Supplementary Figure 10 | Structure of $Li_{2/3}FePO_4$. **a**, Monoclinic unit cell of $Li_{2/3}FePO_4$ reported by Nishimura et al[1]. showing arrangement of low-occupancy and high-occupancy Li columns viewed down $[001]_m$, with occupancy factors of Li1, Li2, Li3 and Li4 sites of 0.4, 0.63, 0.82 and 0.68, respectively. **b**, **S**tructure model of $Li_{2/3}FePO_4$ proposed by Boucher et al.[2] in which occupancy factors of Li1 and Li2 sites have been set to 0 and those of Li3 and Li4 sites have been set to 1.

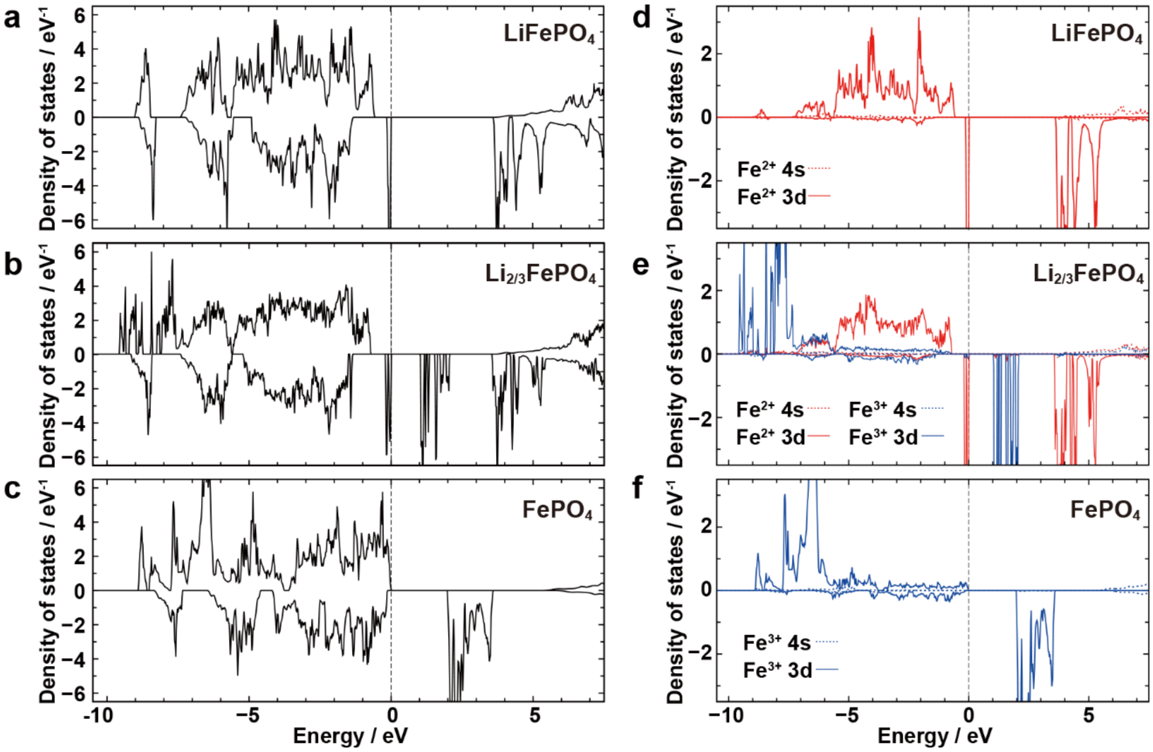

Supplementary Figure 11 | Calculated total density of states (a-c) and partial density of states of Fe ions (d-f) in lithiated, partially delithiated and fully delithiated phases. **a**,**d**, $LiFePO_4$; **b**,**e**, $Li_{2/3}FePO_4$; **c**,**f**, $FePO_4$. Energy levels of the highest occupied states are set at 0 eV on the horizontal axes.

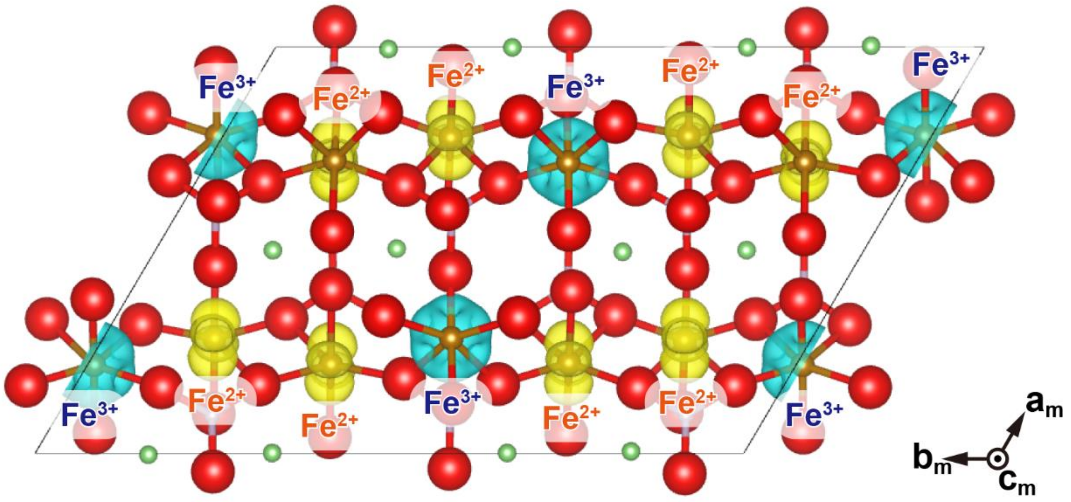

Supplementary Figure 12 | Isosurfaces of norms of band-decomposed wave functions corresponding to the valence band top (yellow) and conduction band bottom (light blue) in $Li_{2/3}FePO_4$. The isovalue of the norm is 0.05 Å^{-3}.

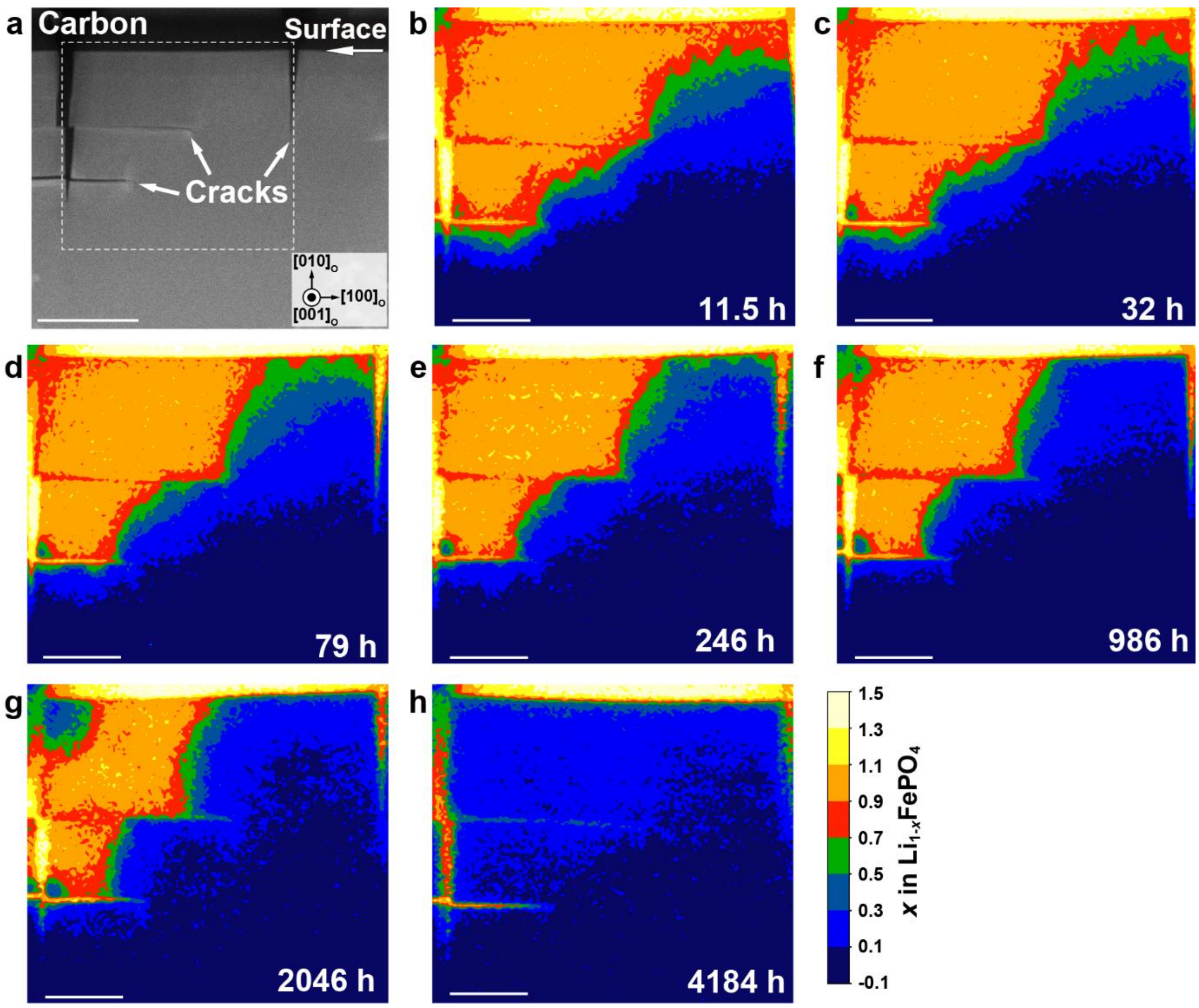

Supplementary Figure 13 | Gradated Li concentration maps corresponding to main text Fig. 5. a, ADF STEM image after delithiation showing cracks perpendicular and parallel to the (010) surface. Scale bar, 200 nm. **b-h**, Maps of the region bounded by the white dashed rectangle in **a** obtained (**b**) 11.5 h, (**c**) 32 h, (**d**) 79 h, (**e**) 246 h, (**f**) 986 h, (**g**) 2,046 h and (**h**) 4,184 h after delithiation. The scale bar to the right of **h** shows colour corresponding to intervals of $\Delta x = 0.2$ for maps **b** to **h**. Scale bar, 100 nm.

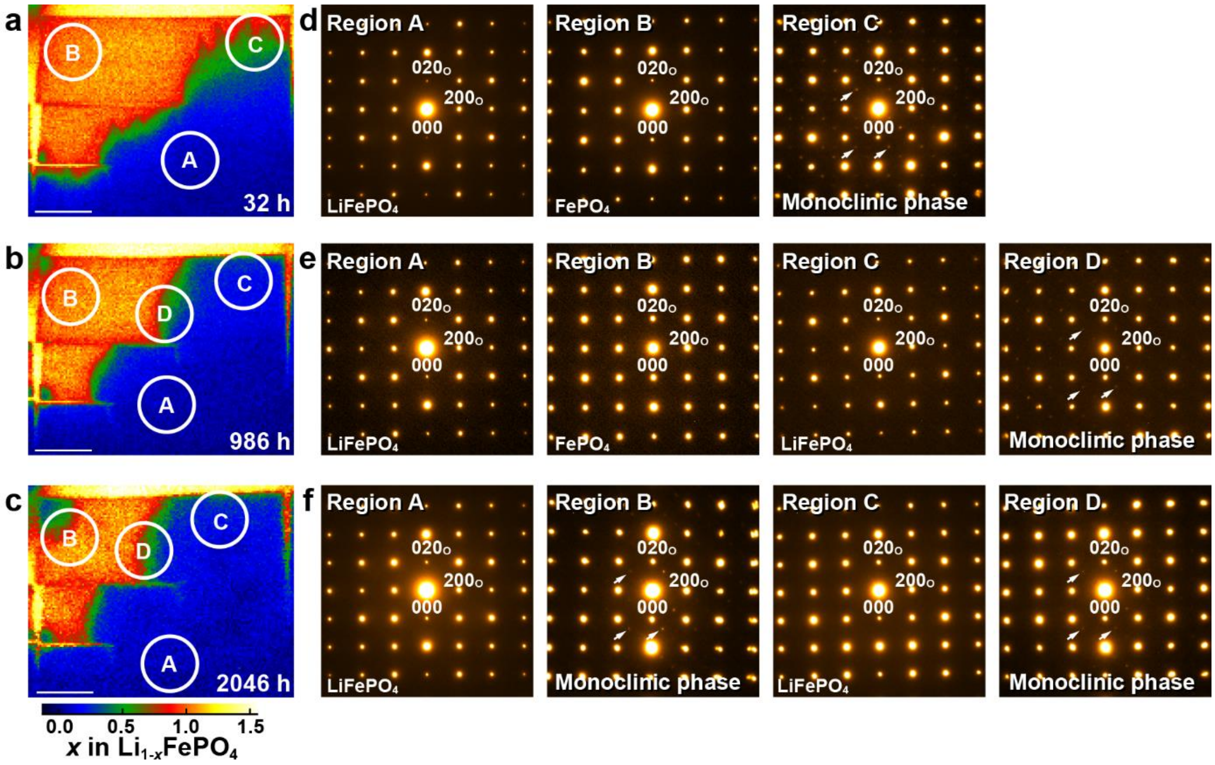

Supplementary Figure 14 | Formation of monoclinic phase at $LiFePO_4$/$FePO_4$ boundaries as a function of time. Li concentration maps of the region bounded by the white dashed rectangle in main text Fig. 5a (**a**) 32 h, (**b**) 986 h and (**c**) 2,046 h after delithiation. Scale bar, 100 nm. **d-f**, Electron diffraction patterns obtained from areas **A**, **B**, **C** and **D** of **a, b** and **c**, respectively. Subscript “o” refers to the orthorhombic structure.

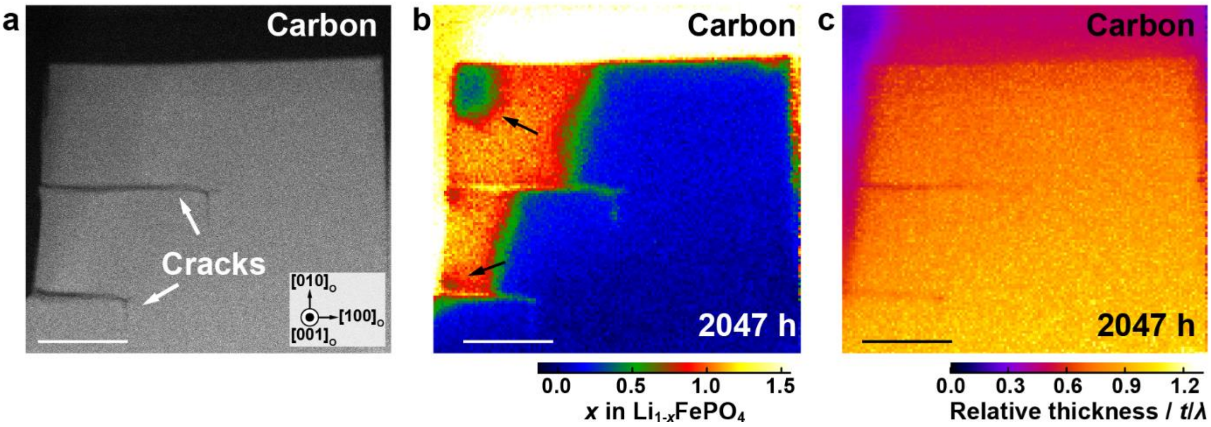

Supplementary Figure 15 | Confirmation of sample stability. a, ADF STEM image of an area of the delithiated sample with minimal electron beam damage. **b**, Li concentration map of the region in **a** obtained 2,047 h after delithiation. The scale bar at the bottom of **b** shows changes in colour as a function of Li content *x*. The black arrows in **b** indicate corner regions to which Li ions returned by external diffusion to form separate regions of intermediate Li content. **c**, A relative thickness (t/λ) map of the region in **a** and **b**. The scale bar at the bottom of **c** shows changes in colour as a function of t/λ. Scale bar, 100 nm.

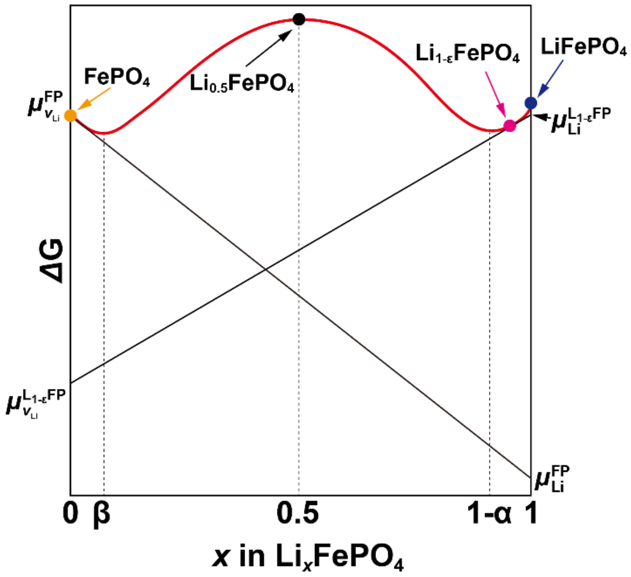

Supplementary Figure 16 | Schematic Gibbs free energy diagram of Li_xFePO_4. The overall Li content of the sample lies to the right of the Li-rich (x = 1-α) phase, so there is a chemical potential difference between delithiated and lithiated regions of the sample that causes Li to diffuse back to the delithiated (surface) regions.

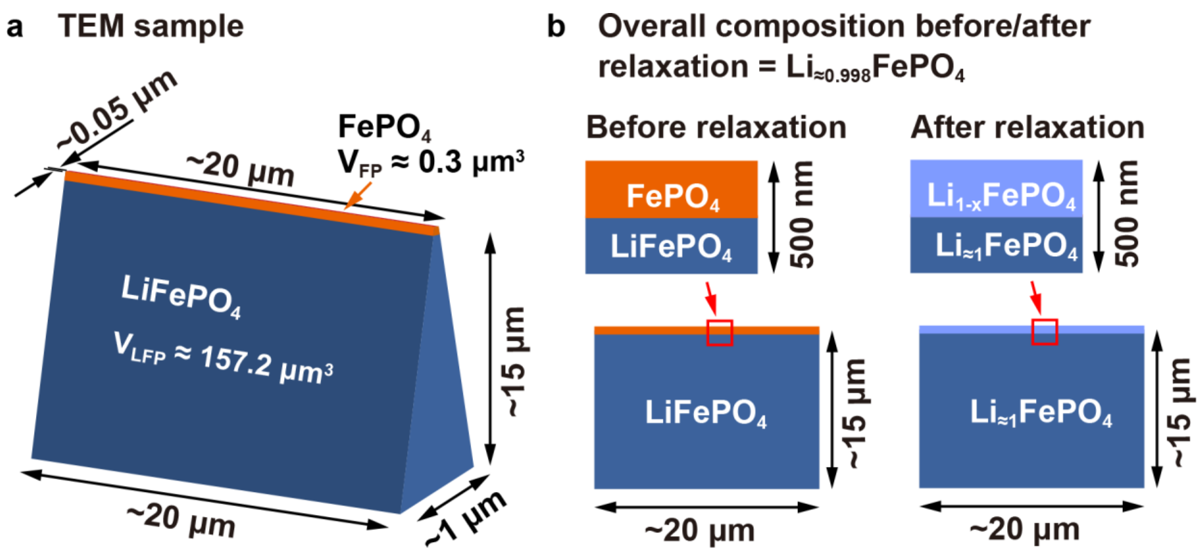

Supplementary Figure 17 | Volumes of Li-poor and Li-rich regions in delithiated crystals. a, Schematic of a delithiated TEM sample with a truncated square pyramidal shape prepared by FIB. **b**, Side views of the sample showing Li-depleted and Li-rich regions before and after relaxation together with magnified views of the surface regions (corresponding to areas imaged by TEM). The volume ratio of Li-poor and Li-rich regions gives an overall composition of $Li_{\approx 0.998}FePO_4$.

Supplementary Table 1. Comparison of calculated and experimental lattice constants of $LiFePO_4$ (*Pnma*), $FePO_4$ (*Pnma*) and $Li_{2/3}FePO_4$ ($P2_1/n$, *c* unique).

	$LiFePO_4$		$FePO_4$		$Li_{2/3}FePO_4$		
	Calc.	Exp. [3]	Calc.	Exp. [3]	Calc.	Calc. [2]	Exp. [1]
a (Å)	10.439	10.3290	9.984	9.8142	11.995	11.9975	11.8389
b (Å)	6.071	6.0065	5.928	5.7893	18.029	18.0396	17.80634
c (Å)	4.742	4.6908	4.885	4.7820	4.794	4.7967	4.73673
γ (°)	-	-	-	-	120.617	120.617	120.4908

Supplementary Note 1. $LiFePO_4$ single crystals and cleaved surfaces before and after delithiation

Supplementary Figure 1 shows SEM micrographs of a cleaved $LiFePO_4$ (010) surface before and after delithiation. The striations correspond to cracks formed at the surface after delithiation perpendicular to the *a* axis (Supplementary Figures. 1c and 1d). Cracks form in this direction because the lattice mismatch between $FePO_4$ and $LiFePO_4$ in the (100) plane (= *bc* plane) is larger than that in the (001) plane. One of the flat areas between cracks in the delithiated sample was isolated by FIB milling and prepared for imaging with a transmission electron microscope.

Supplementary Note 2. Quantitative analysis using valence EELS spectra of $Li_{1-x}FePO_4$

Supplementary Figure 2a shows an Li concentration map of the crystal after delithiation generated using valence electron energy loss spectroscopy (EELS). Supplementary Figure 2b shows spectra from regions **A** (amorphous carbon), **B** ($FePO_4$) and **C** ($LiFePO_4$) in Supplementary Figure 2a. Strong peaks in the case of $FePO_4$ and amorphous carbon, indicated by the arrow in Supplementary Figure 2b, were observed corresponding to interband transitions in $FePO_4$ and the π plasmon peak of amorphous carbon. The $FePO_4$ interband transitions result from electrons moving from states at the top of the valence band, mostly oxygen *p* states, to the mainly Fe 3*d* conduction band, with a change in occupancy from $3d^6$ in $LiFePO_4$ to $3d^5$ in $FePO_4$[4,5]. Li concentrations were estimated from the peak intensities of the spectra. The integration intensity $\boldsymbol{I_\alpha}$ was defined as a 1 eV-wide region beneath the peak, namely the region from 4 to 5 eV, as shown schematically in Supplementary Figure 2c. Integrated $\boldsymbol{I_\alpha}$ peak intensities of $FePO_4$ and $LiFePO_4$ were normalised to 1 and 0, respectively, for x in $Li_{1-x}FePO_4$. In this case, the integrated $\boldsymbol{I_\alpha}$ peak intensity of amorphous carbon exceeds that of $FePO_4$, meaning that it appears as $x > 1$ on the intensity scale even though it contains no Li.

The integrated peak intensity $\boldsymbol{I_\alpha}$ and mean Fe valence states exhibit a linear relationship, meaning that measurement of peak intensities can be used to estimate Li distributions in $Li_{1-x}FePO_4$[6]. Although delocalization and specimen thickness effects, especially around the energy loss regions, make it difficult to determine the precise relationship between the Fe valence state and peak intensity, this method is accurate enough to allow comparison of Li contents in $LiFePO_4$ and $FePO_4$ to be compared with that in the intermediate phase.

A magnified view of part of Supplementary Figure 2a and its EELS spectra are compared in Supplementary Figure 3 to demonstrate the relationship between Li concentration and the change in EELS spectra across the boundary region. Supplementary Figure 3b shows a plot of relative thickness t/λ at each of the numbered points along the dashed line in Supplementary Figure 3a. The variation of the relative thickness t/λ in this part of the sample was small (within ± 0.1) even though the thickness of the sample gradually

increased with distance from the surface. This confirms that the relationship between the Li concentration and thickness is weak in this part of the crystal. Results from quantitative analysis of the spectra are consistent with the change of peak shape of Fe $L_{2,3}$, although it is difficult to estimate the valence state of Fe ions from Fe $L_{2,3}$ edges because of the poor signal-to-noise ratio of the L_2 edge. When a longer exposure time was used to obtain a good signal-to-noise ratio for recording each Fe $L_{2,3}$-edge line-spectrum point, the Fe valence states calculated from Fe $L_{2,3}$ ratios[7] in Li-rich regions indicated significant Li loss from the crystal as a result of excessive electron beam irradiation. Acquisition times for valence EELS spectra used in this study were over 100 times faster than those for Fe $L_{2,3}$-edge EELS spectra, so electron beam damage was greatly reduced. Because of this short recording time, the valence EELS method is considered a more efficient means of quantifying Li distributions in $Li_{1-x}FePO_4$.

Supplementary Note 3. Lattice parameters of $Li_{1-x}FePO_4$

Supplementary Figures 4a and 4b show ADF STEM and Li concentration maps obtained from the crystal after delithiation. Electron diffraction patterns obtained from $FePO_4$ (Supplementary Figure 4c) and $LiFePO_4$ (Supplementary Figure 4d) regions correspond to orthorhombic structures. The as-recorded electron diffraction pattern of the intermediate phase of Supplementary Figure 4e shows extra spots of weak intensity corresponding to a monoclinic phase. To render the extra spots more visible, enhanced and coloured electron diffraction patterns are used in the main text. Supplementary Figure 4f shows the electron diffraction pattern obtained from a mixed two-phase region, region **D**, in Supplementary Figure 4b. Spots produced by a lattice with lattice parameters between those of end-member phases $FePO_4$ and $LiFePO_4$ are visible. In order to confirm the relationship between intermediate phase and end-member phases, a composite pattern comprising the diffraction patterns of $FePO_4$ (region **A**: green), $LiFePO_4$ (region **B**: blue) and intermediate phase (region **C**: red) was prepared as shown in Supplementary Figure 4g. Spots from the $Li_{1-x}FePO_4$ phase appear between those of the end-member phases, confirming that the intermediate phase (or phases) has lattice parameters intermediate to those of the main phases.

Supplementary Note 4. Investigation of ordering of Li-poor columns using ABF STEM

Supplementary Figure 7a shows an integrated ABF STEM image of the monoclinic phase from main text Fig. 3a. Dark spots on a bright background show positions of atom columns. Qualitative determination of the positions of Li-poor columns is possible but quantitative determination of occupancy factors from the contrast is difficult because the signals include coherently scattered electrons. To aid analysis of the experimental profiles, image simulations were performed using the crystal model reported by Nishimura et al.[1] for monoclinic $Li_{2/3}FePO_4$. Intensity line profiles obtained from experimental and simulated images over five regions, labelled 1 to 5 and enclosed by white dashed lines, are shown in Supplementary Figures 7b and 7c, respectively. The intensities of $Li_{0.38}$ (Li-poor) columns are seen to be much brighter than those of $Li_{0.82}$ (Li-rich) columns in the case of lines 1, 3 and 5, and the agreement between experiment and simulation for these three lines is excellent. In contrast, the intensity profiles of lines 2 and 4 differ somewhat between

experiment and simulation. In the intensity profile from the experimental image, the $Li_{0.63}$ columns in lines 2 and 4 lines are brighter than the $Li_{0.68}$ columns, while for the simulation image they are almost the same because their occupancy factors are almost the same. There are several possible reasons for this difference. One is that there is some artefact contrast from ABF imaging, as ABF STEM contrast is very sensitive to measurement conditions[8]. Another possibility is that the actual concentration of Li vacancies is different to that used in the structure model. As the intermediate phase in the real material is subject to a large local strain field, the concentration of Li ions in particular columns may vary. Regardless, the present results confirm that Li-poor columns in the monoclinic phase in the boundary region are ordered.

Supplementary Note 5. Distribution of the monoclinic phase in the delithiated crystal

To determine the distribution of the monoclinic phase in the interface region of the delithiated crystal, data was extracted from BF STEM images using a two-dimensional Fourier transform technique[9]. Supplementary Figure 8a shows an ABF STEM image 53 h after delithiation, and Supplementary Figure 8b shows a BF STEM image of the region in the dashed rectangle in Supplementary Figure 8a. Supplementary Figure 8c shows a two-dimensional Fourier transform of the BF image in Supplementary Figure 8b. Spectral frequencies related to Li-poor ($x \approx 1$), Li-rich ($x < 0.7$) and monoclinic phases are readily distinguished, making it possible to extract data from each phase separately[9]. Images of the phases generated by extracting data from the group of Li-poor, Li-rich and monoclinic spectral frequencies are shown in Supplementary Figures 8d, 8e and 8f, respectively.

The distribution of the monoclinic phase relative to the end-member phases can be better appreciated from the composite of the Li-poor (Supplementary Figure 8d) and monoclinic (Supplementary Figure 8f) phase images shown in Supplementary Figure 9b. Compared with the Li concentration map in Supplementary Figure 9a, the region of the $FePO_4$ phase in Supplementary Figure 9b appears small because images were acquired at different times after delithiation. Nevertheless, the monoclinic phase can be seen to be located near to the Li-poor (i.e., $FePO_4$) phase and forms a facetted interface. The Li content of the monoclinic phase ideally is $x \approx 0.33$ (i.e., composition $Li_{2/3}FePO_4$), but the measured contents correspond roughly to $0.3 < x < 0.7$, implying that monoclinic and other phases, such as $FePO_4$, $LiFePO_4$ or phases with intermediate Li contents and disordered Li vacancies, exist along the observation direction.

Supplementary Note 6. Crystal structures and electronic states of $LiFePO_4$, $FePO_4$ and monoclinic $Li_{2/3}FePO_4$

Supplementary Figure 10 compares the monoclinic structure of $Li_{2/3}FePO_4$ (space group $P2_1/n$ in non-standard setting with unique axis c) obtained experimentally[1] and the model used for DFT calculations[2]. The experimental structure contains four crystallographically distinct Li sites with different occupancy factors. Sites Li1 and Li2 both have low occupancy factors and are aligned in the a_m direction when viewed down the c_m axis; similarly Li3 and Li4 sites have high occupancy factors and are aligned in the same direction, as indicated by dashed boxes in Supplementary Figure 10a. For DFT calculations, the simplified model of the $Li_{2/3}FePO_4$ structure reported by Boucher et al.[2] was used, with low-occupancy sites fully vacant, and

high-occupancy sites fully occupied, as illustrated in Supplementary Figure 10b.

In Supplementary Table 1, the calculated lattice constants of $LiFePO_4$, $FePO_4$ and $Li_{2/3}FePO_4$ are compared with experimental values. The calculated values are about 1 ~ 2% larger than those from experiment[3], within the typical accuracy of DFT calculations using the GGA method for treating exchange-correlation interactions and in good agreement with Boucher et al.'s original report[8]. This model is thus able to reproduce the experimental structure well, despite its simplification in terms of number of distinct Li sites and their partial occupancies. This suggests that the crystallographic symmetry of $Li_{2/3}FePO_4$ is largely determined by ordering of vacancies in the b_m direction, parallel to the direction of Li migration.

Bader charge analysis[10] revealed that Fe ions can be classified into two types according to their net magnetic moment within their Bader volumes. Four Fe ions located between two Li vacancies located at Li1 and Li2 sites on the (010) and (020) planes have 4.30 μ_B. The net magnetic moment of each of the other eight Fe ions is 3.77 μ_B. Valence configurations of Fe^{3+} and Fe^{2+} with 6-fold coordination in high-spin states are $3d^5$ (↑↑↑↑↑) and $3d^6$ (↑↑↑↑↑↓), respectively, showing that Fe^{3+} has a larger magnetic moment than Fe^{2+}. Thus, the first type of Fe ion can be identified as Fe^{3+} and the second type as Fe^{2+}. Holes formed by removal of electrons during delithiation thus appear to be most stable associated with Fe ions neighbouring Li vacancies, at least at 0 K.

Supplementary Figure 11 shows total densities of states and partial densities of states of Fe ions in $LiFePO_4$, $FePO_4$ and $Li_{2/3}FePO_4$. Atom- and orbital-projected densities of states were calculated using the LOBSTER program[11,12]. These give calculated band gaps for $LiFePO_4$ and $FePO_4$ of 3.6 eV and 1.9 eV, respectively, in good agreement with the literature[13–15]. Band gaps in $LiFePO_4$ and $FePO_4$ correspond to occupied and unoccupied Fe 3*d* orbitals. The band gap of $Li_{2/3}FePO_4$ is 1.0 eV, which is smaller than both that of $LiFePO_4$ and that of $FePO_4$, rather than intermediate to them.

As seen in Supplementary Figure 11e, partial densities of states of the transition metals in $Li_{2/3}FePO_4$ correspond to two types of Fe ions, Fe^{2+} and Fe^{3+}. Wave functions derived from electron densities in the vicinity of the valence band top and conduction band bottom in $Li_{2/3}FePO_4$ are localised on Fe^{2+} and Fe^{3+} ions, respectively (Supplementary Figure 12). $Li_{2/3}FePO_4$ can be thought of as the $LiFePO_4$ parent phase with a high concentration of Fe^{3+} defects. In this case, the smaller band gap results from unoccupied Fe^{3+} defect states appearing due to partial delithiation in the gap region of $LiFePO_4$. Similarly, $Li_{2/3}FePO_4$ can be thought of as the $FePO_4$ parent phase with a high concentration of Fe^{2+} defects formed by Li insertion, in which case the smaller band gap results from occupied Fe^{2+} defect states in the gap region of $FePO_4$.

Supplementary Note 7. Effect of electron beam irradiation on crystal integrity and STEM observations

The stability of the TEM sample to electron beam irradiation was checked carefully, as Boucher et al.[8] reported that the monoclinic phase in particular is sensitive to beam damage. Supplementary Figure 14 shows electron diffraction patterns obtained 32 h, 986 h and 2046 h after delithiation. Despite taking Li concentration measurements several times over the same region, the monoclinic phase still appeared in the biphasic boundary region. These results confirm that the conditions used for obtaining Li concentrations from valence EELS measurements (particularly the fast recording time) did not cause undue damage to the crystal.

Nevertheless, it is still possible that the beam caused some Li ions to migrate around the edge of the crystal (main text Fig. 5g). To confirm that electron beam damage had not occurred at the edge of crystal, a Li concentration map was obtained from a region that had barely been exposed to the electron beam beforehand. The results are shown in Supplementary Figure 15. The concentration map, especially the presence of higher Li concentrations at some crystal edges, was essentially the same as that taken 2046 h after delithiation (main text Fig. 5). This gives us confidence that electron beam damage was minimal. Supplementary Figure 15c also shows a map of relative thickness, t/λ, of the region in Supplementary Figure 14b. The thickness gradually increased from around 80 to 105 nm with increasing distance from the surface, meaning that the region was thicker than that of the sample imaged in main text Fig. 5. We thus conclude that Li-ion migration to crystal edges was not a result of electron beam damage or differences in sample thickness, and is more likely due to Li migration across the free surface regions.

Supplementary Note 8. Chemical potentials of $LiFePO_4$ and $FePO_4$

The large difference in chemical potential between the Li-rich and Li-poor regions in chemically oxidised samples can be better appreciated by considering the Gibbs energy diagram in Supplementary Figure 16. If the Li content in Li_xFePO_4 is x = 0.5, the chemical potential of Li in Li-rich ($Li_{1-\alpha}FePO_4$, $\alpha < 0.2$) and Li-poor ($Li_\beta FePO_4$, $\beta < 0.2$) phases is the same, so the material will phase separate at thermodynamic equilibrium to minimise the Gibbs free energy. In our single-crystal sample, however, only a very narrow region of the surface was delithiated, with the majority of the crystal remaining lithiated. Based on estimates of the volumes of the delithiated and lithiated regions of the crystal (described in more detail in Supplementary Note 9), the overall Li deficiency was determined to be ≈0.002, giving a composition well within the stability region of the Li-rich phase of the Gibbs free-energy diagram in Supplementary Figure 16. The chemical potential of Li in the Li-rich phase is higher than that in the Li-poor phase, providing a driving force for Li to diffuse back to the delithiated (surface) regions.

Supplementary Note 9. Schematic diagram of TEM sample before and after Li relaxation

The volumes of $LiFePO_4$ and $FePO_4$ regions in a STEM sample were estimated to be around 157.2 μm^3 and 0.3 μm^3, respectively, assuming the square truncated pyramid configuration shown in Supplementary Figure 17. The overall Li deficiency of the TEM sample after delithiation was thus only 0.2 %, i.e., the overall composition of the sample was $Li_{\approx 0.998}FePO_4$, which can be assumed to remain the same before and after relaxation.

Supplementary References

1 Moreau, P., Mauchamp, V., Pailloux, F. & Boucher, F. Fast determination of phases in Li_xFePO_4 using low losses in electron energy-loss spectroscopy. *Appl. Phys. Lett.* **94**, 123111 (2009).

2 Kinyanjui, M. K. *et al.* Origin of valence and core excitations in $LiFePO_4$ and $FePO_4$. *J. Phys.: Condens. Matter* **22**, 275501 (2010).

3 Kobayashi, S., Fisher, C. A. J., Kuwabara, A., Ukyo, Y. & Ikuhara, Y. Quantitative analysis of Li distributions in battery material $Li_{1–x}FePO_4$ using Fe $M_{2,3}$-edge and valence electron energy loss spectra. *Microscopy* **66**, 254–260 (2017).

4 van Aken, A. P. & Liebscher, B. Quantification of ferrous/ferric ratios in minerals: new evaluation schemes of Fe L_{23} electron energy-loss near-edge spectra. *Phys. Chem. Miner.* **29**, 188–200 (2002).

5 Nishimura, S., Natsui, R. & Yamada, A. Superstructure in the metastable intermediate-phase $Li_{2/3}FePO_4$ accelerating the lithium battery cathode reaction. *Angew. Chem. Int. Ed.* **54**, 8939–8942 (2015).

6 Findlay, S. D., Lugg, N. R., Shibata, N., Allen, L. J. & Ikuhara, Y. Prospects for lithium imaging using annular bright field scanning transmission electron microscopy: a theoretical study. *Ultramicrosc.* **111**, 1144–1154 (2011).

7 Kobayashi, S. *et al.* Simultaneous visualization of oxygen vacancies and the accompanying cation shifts in a perovskite oxide by combining annular imaging techniques. *Appl. Phys. Lett.* **100**, 193112 (2012).

8 Boucher, F., Gaubicher, J., Cuisinier, M., Guyomard, D. & Moreau, P. Elucidation of the $Na_{2/3}FePO_4$ and $Li_{2/3}FePO_4$ intermediate superstructure revealing a pseudouniform ordering in 2D. *J. Am. Chem. Soc.* **136**, 9144–9157 (2014).

9 Andersson, A. S., Kalska, B., Haggstrom, L. & Thomas, J. O. Lithium extraction/insertion in $LiFePO_4$: an X-ray diffraction and Mossbauer spectroscopy study. *Solid State Ionics* **130**, 41–52 (2000).

10 Tang, W., Sanville, E. & Henkelman, G. A grid-based Bader analysis algorithm without lattice bias. *J. Phys.: Condens. Matter* **21**, 084204 (2009).

11 Maintz, S., Deringer, V. L., Tchougreeff, A. L. & Dronskowski, R. LOBSTER: A tool to extract chemical bonding from plane-wave based DFT. *J. Comput. Chem.* **37**, 1030–1035 (2016).

12 Maintz, S., Deringer, V. L., Tchougréeff, A. L. & Dronskowski, R. Analytic projection from plane-wave and PAW wavefunctions and application to chemical-bonding analysis in solids. *J. Comput. Chem.* **34**, 2557–2567 (2013).

13 Zhou, F., Kang, K., Maxisch, T., Ceder, G. & Morgan, D. The electronic structure and band gap of $LiFePO_4$ and $LiMnPO_4$. *Solid State Commun.* **132**, 181–186 (2004).

14 Zaghib, K., Mauger, A., Goodenough, J. B., Gendron, F. & Julien, C. M. Electronic, optical, and magnetic properties of $LiFePO_4$: Small magnetic polaron effects. *Chem. Mater.* **19**, 3740–3747 (2007).

15 Chevrier, V. L., Ong, S. P., Armiento, R., Chan, M. K. Y. & Ceder, G. Hybrid density functional calculations of redox potentials and formation energies of transition metal compounds. *Phys. Rev. B*

82, 075122 (2010).
